# Synergetic Effect of β-Cyclodextrin and Its Simple Carbohydrate Substituents on Complexation of Folic Acid and Its Structural Analog Methotrexate

**DOI:** 10.3390/pharmaceutics16091161

**Published:** 2024-09-03

**Authors:** Magdalena Ceborska, Aleksandra Siklitskaya, Aneta Aniela Kowalska, Karolina Kędra

**Affiliations:** 1 Faculty of Mathematics and Natural Sciences, Cardinal Stefan Wyszyński University, Wóycickiego 1/3, 01-938 Warsaw, Poland; 2 Institute of Physical Chemistry, Polish Academy of Sciences, Kasprzaka 44/52, 01-224 Warsaw, Poland; asiklit@ichf.edu.pl (A.S.); akowalska@ichf.edu.pl (A.A.K.)

**Keywords:** antifolate, cyclodextrin, supramolecular chemistry, differential scanning calorimetry, molecular modeling

## Abstract

Folic acid (FA) and its structural analog, anticancer medicine methotrexate (MTX), are known to form host/guest complexes with native cyclodextrins, of which the most stable are formed with the medium-sized β-cyclodextrin. Based on our research, proving that simple sugars (D-glucose, D-galactose, and D-mannose) can form adducts with folic acid, we envisioned that combining these two types of molecular receptors (cyclodextrin and simple carbohydrates) into one may be beneficial for the complexation of FA and MTX. We designed and obtained host/guest inclusion complexes of FA and MTX with two monoderivatives of β-cyclodextrin—substituted at position 6 with monosaccharide (glucose, G-β-CD) and disaccharide (maltose, Ma-β-CD). The complexation was proved by experimental (NMR, UV-vis, IR, TG, DSC) and theoretical methods. We proved that derivatization of β-cyclodextrin with glucose and maltose has a significant impact on the complexation with FA and MTX, as the addition of one glucose subunit to the structure of the receptor significantly increases the value of association constant for both FA/G-β-CD and MTX/G-β-CD, while further extending a pendant chain (incorporation of maltose subunit) results in no additional changes.

## 1. Introduction

It is known that drug delivery systems based on the inclusion complexation of drug molecules with cyclodextrins (CDs) can improve their bioavailability and reduce toxicity and side effects. Such host/guest complexes increase guest molecules’ stability and water solubility and enable their targeted delivery [1]. Inclusion complexes based on unsubstituted cyclodextrins can, to some extent, improve the drug delivery to the diseased tissues. However, there is a need to search for non-toxic substituents modifying CDs to enhance the targeted delivery of incorporated molecules. CDs are macrocyclic oligosaccharides built from 1,4-linked glucose units possessing hydrophobic cavity. They enable the complexation of a wide range of organic molecules and hydrophilic outer space and lead to their good water solubility. They are used as pharmaceutical excipients in clinical practice and are considered safe. They are widely used for complexing bio-relevant molecules in solution [2,3,4,5] and in the solid state [6]. 

**Figure 1:**
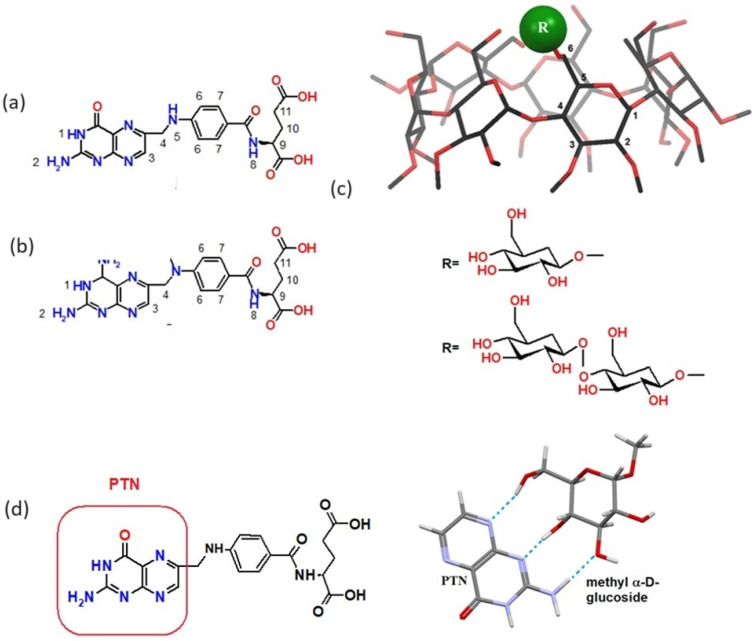
Molecular structures of guests: FA (**a**) and MTX (**b**) and host cyclodextrins: G-β-CD and Ma-β-CD, with atoms numbering, (**c**). Molecular recognition of pterin (PTN) part of folic acid by methyl α-D-glucoside (**d**), (PTN subunit is highlighted in the structure of FA).

FA (vitamin B9, Figure 1a) is a synthetic, oxidized form of folate. It plays a pivotal role in many metabolic and biochemical processes in the human body. It is responsible for biosynthesis of nucleotides and is fundamental for nucleic acid metabolism, cell division, and gene expression [7]. FA is often used as a targeting part in prodrugs and drug carriers aimed at folate receptors [8,9,10], as well as in more sophisticated drug delivery systems for anticancer therapies [11,12,13], owing to its uptake by folate receptor-positive cancer cells. 

MTX (Figure 1b) is an example of antifolate, which is structurally similar to FA. MTX differs from FA with the substituent on C4 carbon of pteridine ring (-NH_2_ instead of the carbonyl group) and substitution of N5 hydrogen with methyl group. Antifolates [14] inhibit the action of vital enzymes (thymidylate synthase, TS; dihydrofolate reductase, DHFR; and glycinamide ribonucleotide formyl transferase, GARFT) and are used as antitumor and antineoplastic agents.

The complexation of FA with native CDs was previously studied [15,16,17,18]. α-CD was too small to form stable complexes with FA/antifolate, whereas β-CD and γ-CD formed pseudorotaxane inclusion structures (*K*_a_ = 131 ± 10.5 for FA/β -CD and 28 ± 3.4 for FA/γ-CD). 

The complexation of MTX with various CDs was also previously studied. Pattarino et al. [19] studied the formation of the complexes of MTX with native cyclodextrins under acidic conditions (pH = 5). As with FA, there was no complexation with the smallest α-CD; weak complexes were formed with the biggest γ-molecule (*K*_a_ = 7 ± 0.9), and the most stable were obtained with β-CD (389.7 ± 104.5). The substitution of β-CD with hydroxypropyl groups leads to the formation of much weaker CD/MTX complexes. Complete substitution of β-CD with methyl groups blocked the complexation process altogether, while substituting 2 and 6 positions led to the complexes of the highest stability (952.1 ± 12.0 M^−1^). 

It is well known that structural modifications of CD rims by different substituents highly affect complex formation. The prediction of host/guest complexes formation in the solution and their stability is challenging due to diverse phenomena while molecular recognition occurs. Driving forces that can contribute to the formation of a complex include electrostatic, van der Waals interactions, hydrogen bonding, etc. In an aqueous solution, the hydrophobic effect also plays an important role [20]. In the case of CD complexes, the enthalpy–entropy compensation phenomenon was also observed, and its impact on the thermodynamic parameters of binding was investigated [20,21,22]. Schönbeck et al. studied the structural modifications of CD rims by different numbers of hydroxypropyl chains, which affect the thermodynamic parameters, increasing the complexation of enthalpies, entropies, and heat capacity [20]. They found that the guest molecule determined the enthalpy and entropy compensation. The compensation phenomena were related to desolvated molecular surface hydrophilicity/hydrophobicity.

Explaining the formation mechanism of individual complexes with substances is complicated and is determined by thermodynamic parameters, a kind of thermodynamic fingerprint for complex formation. Despite long studies, the origin of the enthalpy–entropy compensation observed for CD complexes still needs to be determined.

Surprisingly, in the course of our research on molecular recognition of FA [15,16,17,18,19] and antifolates [23] by α-, β-, and γ-CDs, it was found that simple sugars such as methyl α-D-glucoside can form associates with FA and antifolate pemetrexed (Alimta). Further studies [24] proved that simple sugars (D-glucose, D-galactose, and D-mannose) and their derivatives, protected at anomeric position, can form complexes with FA. The quantum-mechanical calculations indicated that such a phenomenon is responsible for forming three very strong and directional hydrogen bonds between the FA pterin part and three free hydroxyl groups of simple sugar, as in the case of forming nucleobase pairs.

The knowledge of the possibility of forming the complexes of FA and antifolates with CDS and simple sugars leads us to conclude that combining these two types of molecular receptors into one may be beneficial for the complexation process of such guest compounds. 

Knowing that, out of three native CDs, β-CD forms the most stable complexes with both FA and MTX, we decided to apply sugar-derived β-CDs to our studies. The CDs of choice were substituted at position 6 with monosaccharide (glucose) and built from two glucose subunits disaccharide (maltose). Moreover, 6-O-α-D-glucosyl-β-cyclodextrin (G-β-CD, Figure 1c) and 6-O-α-D-maltosyl-β-cyclodextrin (Ma-β-CD, Figure 1c) are the derivatives of native β-CD of similar cavity size but with better water solubility [25,26,27,28].

Herein, FA- and MTX host/guest complexes with G-β-CD and Ma-β-CD in solution (UV-vis, ^1^H NMR) and solid state (TG, DSC, FTIR–ATR) were studied. The computational approach for structural elucidation of the obtained complexes was also applied.

## 2. Materials and Methods

### 2.1. Materials

FA (was obtained from Sigma Aldrich; MTX-Na_2_ (98%), G-β-CD (98%), and Ma-β-CD from ABCR (98%); β-CD from Cyclolab, D_2_O from Eurisotop, and used as received. For aqueous solutions, HPLC purity grade H_2_O was used. 

### 2.2. Preparation of Solid-State Complexes

To the solution of MTX sodium salt in water (49.8 mg (0.1 mmol), 1 equiv per 2 mL) or FA disodium salt in water (44.1 mg (0.1 mmol), 1 equiv per 2 mL H_2_O), a solution of G-β-CD in water (129.7 mg (0.1 mmol), 1 equiv per 8 mL H_2_O) was added and thus 1:1 obtained solution was stirred at RT for 1 h. The solvent was evaporated, and the resulting solid was dried under a vacuum. The procedure was repeated for Ma-β-CD using 145.9 mg (0.1 mmol) of Ma-β-CD. 

### 2.3. Methods

#### 2.3.1. Ultraviolet–Visible Spectroscopy (UV-Vis)

The UV–visible absorption spectra of FA, MTX, and their respective associates with G-β-CD and Ma-β-CD were recorded using Evolution 220 UV/VIS spectrometer (Thermo Scientific, Waltham, MA, USA) at 25 °C in the range 250–450 nm. All measurements were performed in phosphate-buffered saline (PBS, pH = 7.4).

- UV-vis titration experiments

Change in absorption of FA and MTX was measured as a function of G-β-CD and Ma-β-CD concentration. The concentration of the guest compound was held constant at 10^−5^ mol/dm^−3^ for both FA and MTX. 

The host concentration was changed from 0 to at least a hundredfold excess (depending on the host solubility in water). All the measurements were performed in phosphate-buffered saline (pH = 7.4). 

The stoichiometry and association constants were calculated using the HypSpec program [29,30,31]. Several binding equilibria between carbohydrate host (H) and guest (G) were considered. The experimental data were either applied to single equilibrium model 1:1 (H:G) or multiple binding equilibria models, such as 1:1 (H:G) + 1:2 (H:G) model or 1:1 (H:G) + 2:1 (H:G) model, for best data fit with HypSpec program according to general rules for determination of association constants from titration experiments in supramolecular chemistry [32,33,34].

#### 2.3.2. NMR Spectroscopy

The ^1^H NMR spectra were recorded on Bruker Avance 400 MHz spectrometer. Spectra of G-β-CD, Ma-β-CD, FA-Na_2_, MTX-Na_2_, and their respective complexes were recorded in D_2_O. Chemical shifts are reported in ppm relative to HDO (δ = 4.8 ppm). 

#### 2.3.3. Molecular Modeling

The initial neutral structures of G-β-CD and Ma-β-CD were derived from the unsubstituted β-CD crystal structure provided by the Cambridge Crystallographic Data Centre [35]. The host and guest molecule structures were primarily optimized using the ORCA software package(Version 5.0) [36], which employed the BP86 functional framework [37] with D3BJ dispersion correction [38,39] to balance cost and accuracy effectively. Geometrical counterpoise correction (gCP) [40,41] was applied to minimize intra- and intermolecular basis set superposition error. The resulting structures underwent numerical second derivative calculations and were confirmed to possess only positive normal modes. 

Subsequently, primer complexes were formed for each “in” and “out” configuration based on the relative position of the guest. These complexes were then subjected to semi-empirical PM3 [42] method-based optimization, followed by the HF-3C Grimme’s 3-corrected Hartree–Fock QM method [43] and the final optimization procedure mentioned in the paragraph above.

#### 2.3.4. Thermal Analysis: Differential Scanning Calorimetry (DSC) and Thermogravimetry (TG) Analysis

The thermal behavior of obtained solid-state complexes and constituent compounds were investigated using a differential scanning calorimeter DSC 3 and thermogravimetric analyzer TGA/DSC 3+ (Mettler-Toledo GmbH, Schwerzenbach, Switzerland). All measurements were performed under an argon atmosphere with a flow rate of 60 mL min^−1^. At the simultaneous TGA/DSC analysis, the sample was placed in a 40 μL aluminum crucible closed with a perforated lid and was heated from 25 to 500 °C with a heating rate of 10 °C min^−1^. The 6 μL aluminum crucible closed with a perforated lid was used at DSC measurements, and the sample was analyzed in temperatures ranging from −80 to 500 °C with a heating rate of 10 °C min^−1^.

#### 2.3.5. Infrared Spectroscopy

FTIR–ATR measurements were performed using a JACSO FT/IR-6200 Spectrometer at 293 K (Jasco, Easton, PA, USA) using a high-resolution Attenuated Total Reflectance (ATR) technique with crystalline ZnSe (reflection angle 40°). The resolution was 4 cm^−1^, and the number of scans was 16.

## 3. Results

### 3.1. UV-Visible Spectroscopy (UV-Vis)

All the UV measurements were carried out in phosphate-buffered saline (PBS) at pH = 7.4. At this pH, both FA [p*K*_a_s for carboxylates: p*K*_a1_ = 2.35, p*K*_a2_ = 4.56 [44], and MTX p*K*a_1_ = 4.8, p*K*a_2_ = 5.5 [45]] exist as dianions. Upon titration, the concentration of the guest molecule remained constant to avoid dilution effects and the influence of a possible guest (FA or MTX) aggregation.

FA shows two absorption maxima at 281 and 350 nm, while in the MTX spectrum, three strong absorption peaks appear at 258, 300, and 372 nm. 

**Figure 2:**
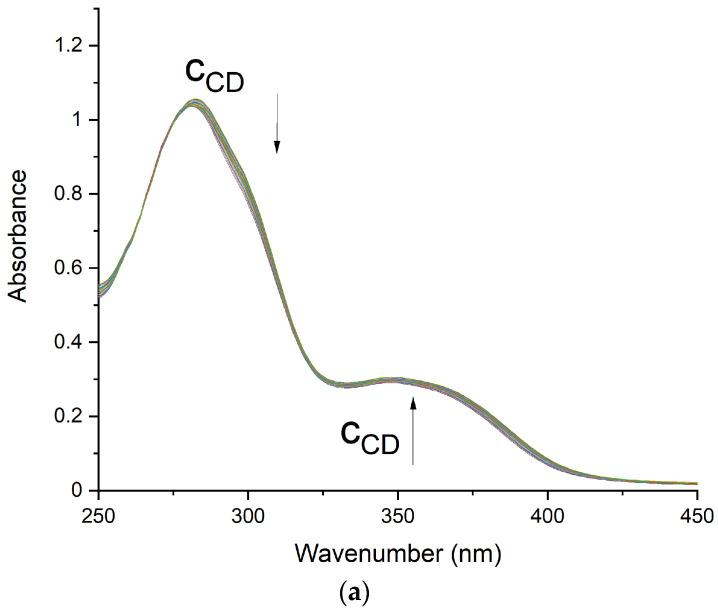

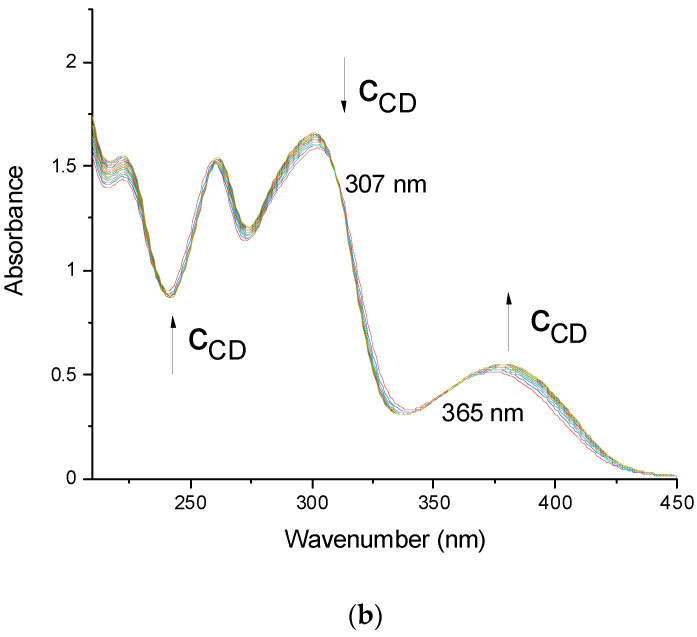
Experimental titration spectra at pH = 7.4 (PBS) for the following: (**a**) FA/G-β-CD; (**b**) MTX/G-β-CD.

The titration experiments were performed to evaluate stoichiometry and association constants for FA and MTX with carbohydrate derivatives of β-CD. (Figure 2a,b and Figures S1 and S2).

For evaluation of the stoichiometry and association constants (*K_a_*s), the obtained data were fitted with the HypSpec program. Because both FA and MTX possess two carboxylate groups in their structure, they tend to form dimers in solution, which is typical for carboxylic acids. Therefore, the FA and MTX dimers’ formation was considered while calculating the stability constants of their respective complexes with G-β-CD and Ma-β-CD. The corresponding titration curves for FA/G-β-CD, FA/Ma-β-CD, MTX/G-β-CD, and MTX/Ma-β-CD were consistent with a 1:1 host/guest binding model (for *K*a values, see Table 1). The general method for analyzing residual distribution in titration data fitting was applied [33,34].

It can be clearly seen from Table 1 that the derivatization of β-CD with glucose and maltose (disaccharide consisting of two glucose subunits) has a huge impact on the complexation with FA and MTX. The addition of one glucose significantly increases the value of the association constant while further extending the pendant chain (incorporation of maltose chain) gives no additional changes and results in maintaining the values of FA/Ma -β-CD and MTX/Ma-β-CD association constants similar to those obtained with G-β-CD. To explain these results, further experiments were performed, including studies in solution (^1^H NMR) and in the solid state (DSC/TG; IR), as well as theoretical calculations.

### 3.2. NMR Spectroscopy

Analysis of the ^1^H NMR spectra of equimolar mixtures of hosts (G-β-CD or Ma-β-CD) and guest molecules (FA or MTX) in D_2_O indicates that changes appear in chemical shifts of host and guest protons. Nevertheless, due to the overlapping signals of the glucose subunits from the macrocyclic core and appended carbohydrate unit (glucose or maltose) of studied cyclodextrins, it is impossible to precisely determine and interpret all host proton signals. Therefore, only changes in guest molecules’ chemical shifts are considered. 

**Figure 3:**
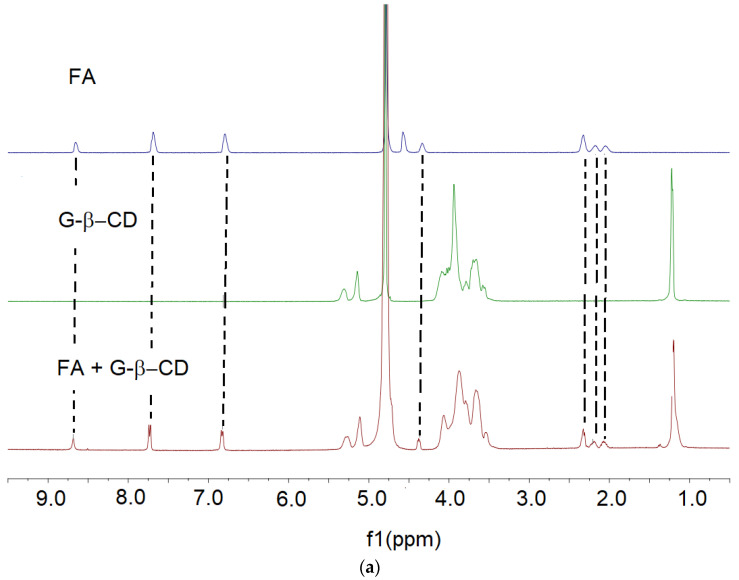

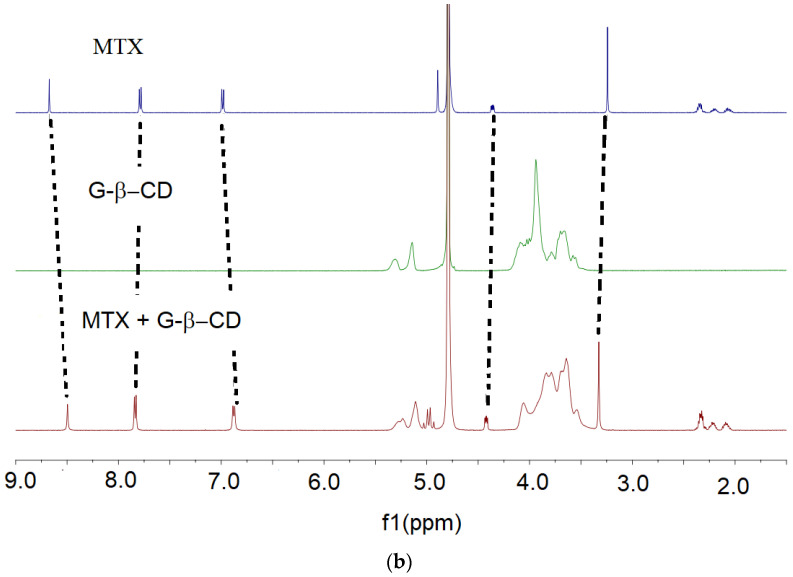
Stacked ^1^H NMR spectra in D_2_O of (from top to bottom): (**a**) FA, G-β-CD, FA/G-β-CD; (**b**) MTX, G-β-CD, MTX/G-β-CD.

**FA/G-β-CD** In the ^1^H spectrum of equimolar mixture of FA and G-β-CD in D_2_O (Figure 3a), the most pronounced changes appear for protons FA#H3, FA#H6, FA#H7, FA#H10A, FA#H10B, and FA#H11 (Δδ being 0.04, 0.05, 0.04, 0.02, 0.05, and 0.07 ppm, Table 2a). From these data, it may be concluded that interactions between FA and CD host (either via the hydrophobic cavity or pendant glucose unit) appear for the bicyclic part of the guest molecule and aromatic (phenyl) subunit and are supported by interactions of FA#H10A, FA#H10B, and FA#H11 protons belonging to the aliphatic chain (with -COOH-γ) of glutamic acid subunit of FA. 

**FA/**Ma**-β-CD** The analysis of the ^1^H NMR spectrum of an equimolar mixture of Ma -β-CD with FA in D_2_O (Figure S7) clearly shows significant changes in chemical shifts assigned to guest protons. The most significant changes appear for protons of FA#H3, FA#H6, FA#H7, FA#H10A, FA#H10B, and FA#H11 (Δδ being 0.05, 0.08, 0.02, and 0.05 ppm, respectively; Table S1). This means that a major part of FA takes part in binding with the CD host. The most significant interactions are identified as these of aromatic parts (both pterin part as well as phenyl part) as well as interactions of aliphatic protons FA#H11 belonging to γ-part (three-carbon-length aliphatic chain) glutamic acid subunit of FA.

**MTX/G-β-CD** (Figure 3b) Similarly, as in the case of FA/G-β-CD, in the ^1^H spectrum of the equimolar mixture of MTX and G-β-CD in D_2_O, the most pronounced changes appear for protons belonging to the bicyclic and phenyl subunit of the guest molecule (MTX#H3, Δδ = 0.17 ppm and protons MTX#H6, MTX#H7 with Δδ = 0.31 and 0.07 ppm, respectively). Additionally, binding is supported by ethylenic H#4 protons and H#5 protons belonging to the methyl group attached to the nitrogen atom. Contrary to the binding pattern of FA, MTX binding with CD molecule is supported by H-interactions of shorter chain (-COOH-α) of glutamate subunit (MTX#H9, Δδ = 0.06 ppm) (Table 2b).

**MTX/**Ma**-β-CD** The ^1^H spectrum of the equimolar mixture of MTX and Ma-β-CD (Figure S8) is very similar to the ^1^H spectrum of the equimolar mixture of MTX and G-β-CD, suggesting the occurrence of a similar binding pattern. The most significant changes appear for bicyclic MTX#H3 (Δδ = 0.25 ppm), ethylenic MTX#H4 (Δδ = 0.13 ppm), methyl MTX#H5 (Δδ = 0.14 ppm), aromatic MTX#H6 (Δδ = 0.19 ppm), MTX#H7 (Δδ = 0.07 ppm), and aliphatic (belonging to the shorter chain of glutamic acid subunit) protons MTX#H9 (Δδ = 0.05 ppm) (Table S2).

### 3.3. Molecular Modeling

All obtained complexes were modeled as inclusion compounds, with two possible orientations of the guest molecule (glutamate part sticking out either from the wider or narrower rim of the used CD). Given the previous knowledge, the first one is more probable; however, in SI, we also present the other one (Figures S15–S18).

**Figure 4:**
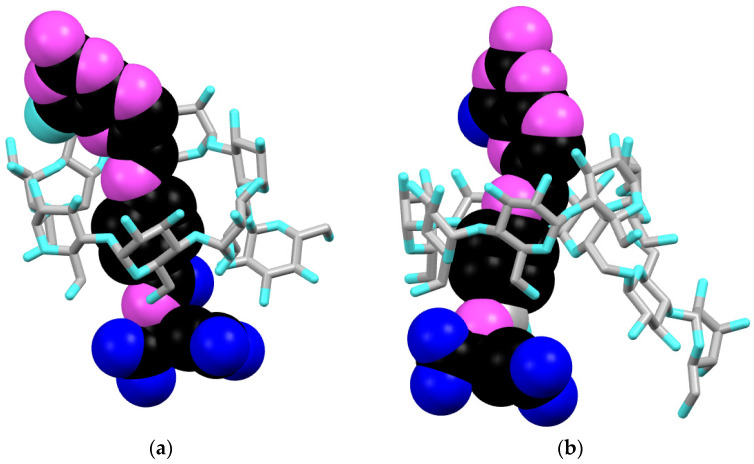

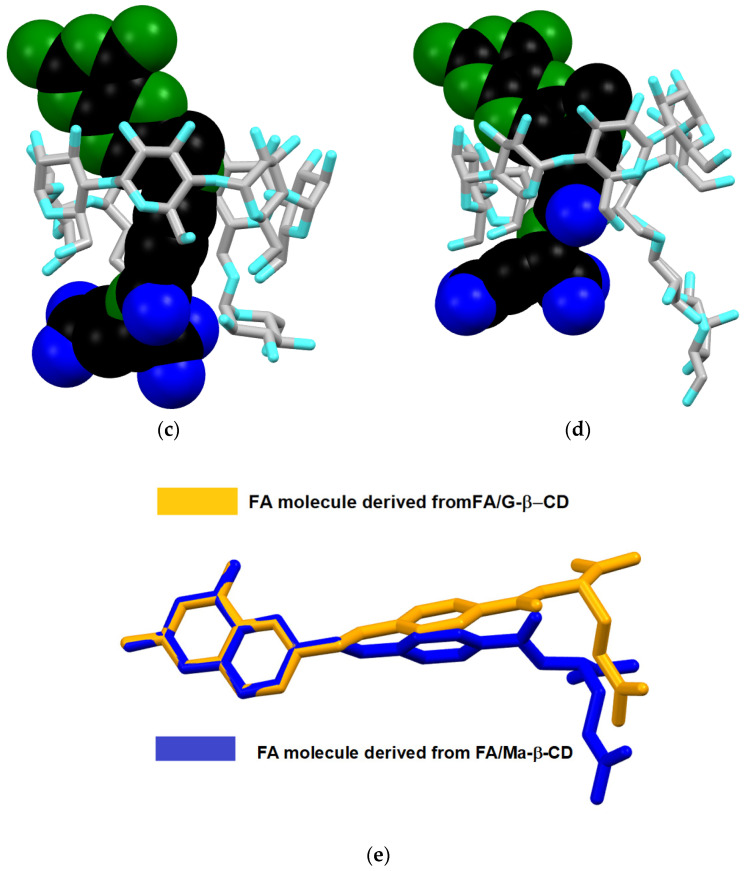
Comparison of the complexes: (**a**) 1:1 (H:G) FA/G-β-CD; (**b**) 1:1 (H:G) FA/Ma-β-CD; (**c**) 1:1 (H:G) MTX/G-β-CD; (**d**) 1:1 (H:G) MTX/Ma-β-CD; (**e**) overlay of the FA guest molecule derived from its complexes with G-β-CD (yellow) and Ma-β-CD (blue).

**FA/G-β-CD** In the 1:1 host: guest inclusion complex of folic acid with G-β-CD phenyl ring of FA is fully encapsulated inside the host cavity, with the bicyclic part of the structure interacting with the secondary side of G-β-CD. In contrast, glutamic acid moiety reaches out from the primary side of the macrocyclic host (Figure 4a and Figure S9). The conformation of FA inside the complex is “twisted” so that it can form more stable complexes with the host CD via additional weak interactions (hydrogen bonds) between C-H groups of the γ-part of the glutamate subunit and oxygen atoms belonging to the primary side hydroxyl groups of G-β-CD and with oxygen atoms belonging to hydroxyl groups of glucose unit attached to the CDs’ primary side. 

**FA/**Ma**-β-CD** In the 1:1 host: guest inclusion complex of FA with Ma-β-CD, similarly to its complex with G-β-CD, the phenyl moiety is placed in the center of CD’s cavity, with pterin subunit interacting with the wider rim of cyclodextrin host and glutamic acid moiety reaching out from the primary side of the macrocyclic host (Figure 4b and Figure S10). Hydrogen bonds between the N-H amidic group and C-H groups of the γ-part of the glutamate FA subunit and oxygen atoms belonging to the primary side hydroxyl groups of G-β-CD additionally stabilize the structure of the associate.

**Comparison of FA/G-β-CD and FA/**Ma**-β-CD** Both complexes exhibit similar geometry and similar binding patterns with only slight changes in the conformation of guest molecule. Figure 4e presents the superposition of FA acid from both complexes, showing the change in FA conformation (FA/G-β-CD—yellow and FA/Ma-β-CD—blue). The angle between the planes comprised pteridine and *p*-nitrobenzoate subunits of FA equal to 54.82° (for FA/G-β-CD) and 89.45° (for FA/Ma-β-CD), respectively, (Figure S11). The data obtained from theoretical calculations fully support previously obtained experimental results: NMR (geometry) and UV-vis (comparable association constants due to the conformational similarity).

**MTX/G-β-CD** In the complex of methotrexate with G-β-CD, which is similar to the complexes obtained for FA guest molecule, the phenyl moiety is placed in the center of CD’s cavity, with pterin subunit interacting with the wider rim of cyclodextrin host and glutamic acid moiety reaching out from the primary side of the macrocyclic host (Figure 4c and Figure S12). The amidic group of glutamate part of MTX interacts with a narrower rim of G-β-CD via N-H···O hydrogen bonds. The conformation of the guest molecule is additionally stabilized by C-H···O and N-H···O interactions between the α-chain of glutamate (shorter chain) and glucose unit attached to the cyclodextrin core.

**MTX/**Ma**-β-CD** In the complex of methotrexate with Ma-β-CD, the *p*-aminobenzoate moiety of the guest molecule is placed tightly in the center of the Ma-β-CD cavity. At the same time, the pteridine ring reaches out from the secondary side of the host molecule, with the glutamate subunit sticking out from the narrower rim (Figure 4d and Figure S13). The conformation of the guest molecule is identical to its conformation inside the complex with G-β-CD (Figure S14). The maltose chain attached to a macrocyclic ring of β-CD does not interact with the gust molecule. However, the conformation of MTX is additionally stabilized by interactions of glutamate subunit with hydroxyl groups of the narrower rim of Ma-β-CD.

**Comparison of MTX/G-β-CD and MTX/**Ma**-β-CD** The conformation of the guest molecule is identical in both of the discussed complexes. Nevertheless, its binding pattern changes slightly due to the differences in the conformation of the guest molecules. These differences are not significant enough to change the overall geometry of the complex and influence its stability (as seen from UV-vis and NMR experiments).

### 3.4. Thermal Analysis

**Figure 5:**
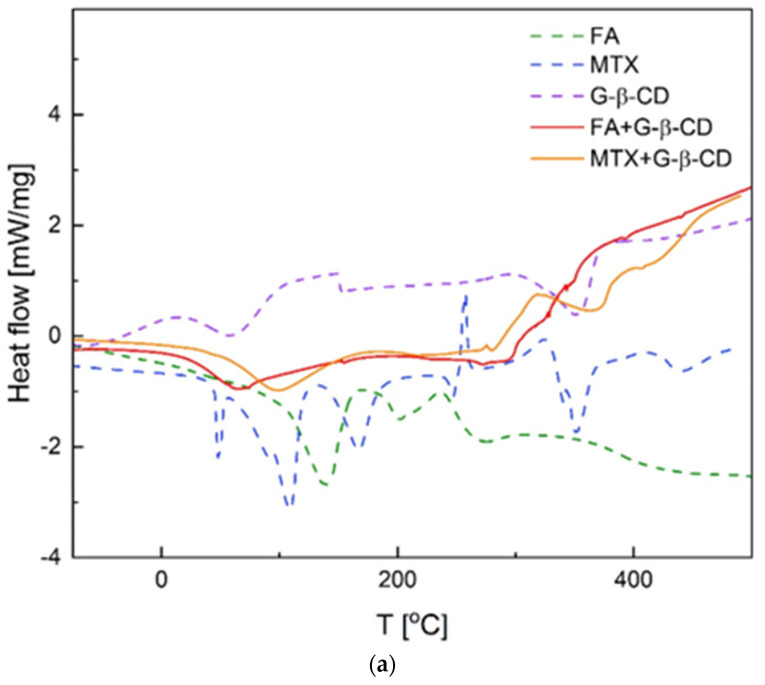

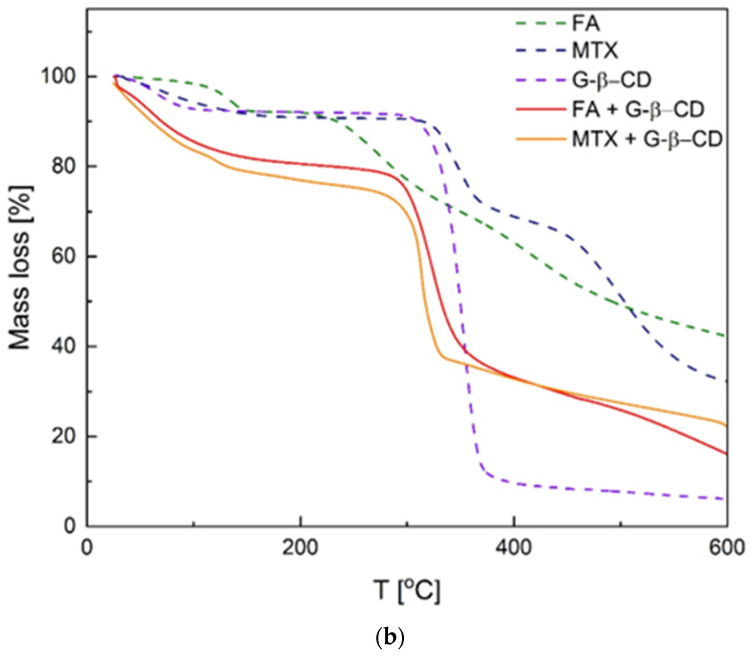
DSC curves (**a**) and TG analysis (**b**) of solid complexes of FA and MTX with G-β−CD and of parent compounds.

The DSC plots (Figure 5a and Figure S19a) and TG curves (Figure 5b and Figure S19b) show the evident change in the thermal properties of the parent compounds compared to the solid-state complexes consisted of modified cyclodextrins with FA and MTX. The change in the thermal behavior confirms the associates’ involvement.

The stages of mass loss resulting from the TG analysis indicate the thermal decomposition of the complexes. The first, below 150 °C, indicates dehydration of the samples. Above this, the complexes decompose (at temperatures 150–500 °C). Then, the inclusion complexes begin to decompose, as seen in the TG curves in the temperature range of 150–500 °C. DSC curves in this temperature range show a series of exothermic peaks resulting from the decomposition of the tested compounds. 

As previously showed, thermal analysis of FA solid-state complexes with α-, β-, and γ-CDs [17] showed exothermic peaks in the range of 200–250 °C and a noticeable loss of sample mass related to the decomposition of the studied compounds. Similar exothermic peaks, which are also related to the mass loss, are revealed at DSC curves of FA and MTX complexes with G-β-CD and Ma-β-CD at slightly higher temperatures above 300 °C (Figure 5a and Figure S19a, respectively). The exothermic effects of the decomposition of complexes and their components are higher in the case of MTX than in FA associates. This effect is the most evident for complexes formed with Ma-β-CD, for which the associates seem more thermally stable.

### 3.5. Infrared Spectroscopy

**Figure 6:**
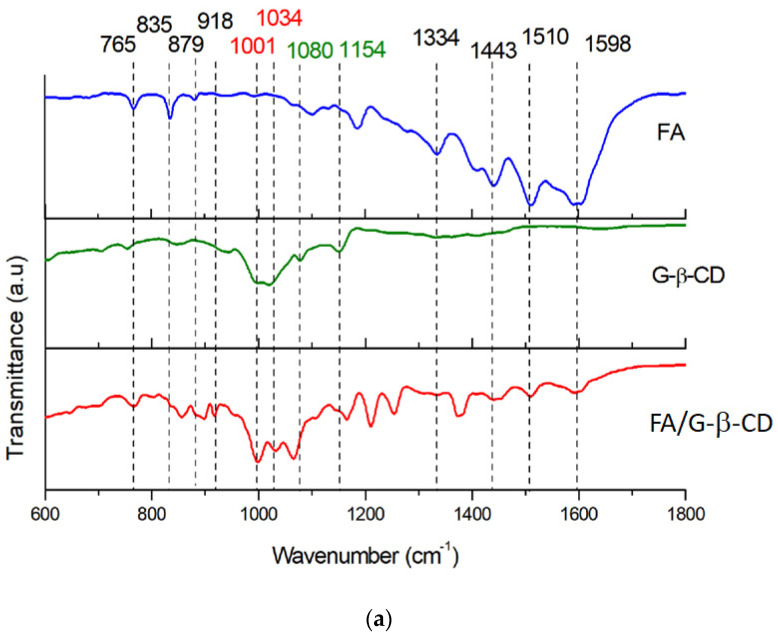

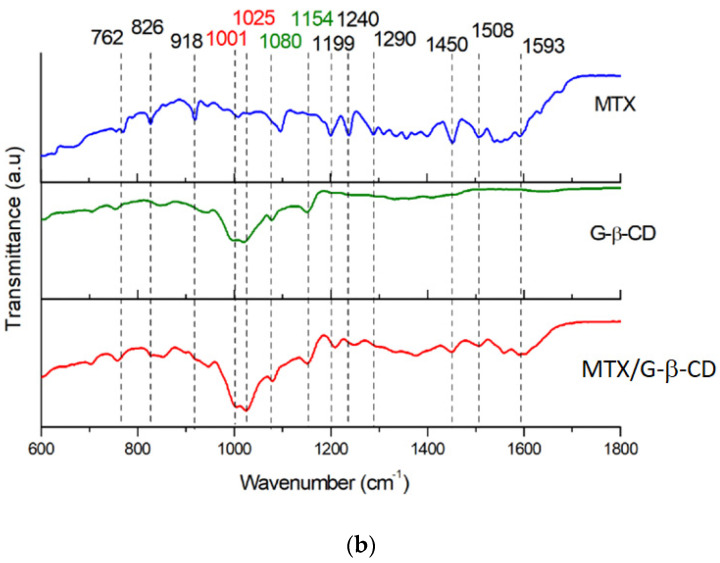
Stacked FTIR spectra: (**a**) FA, G-β-CD, FA/G-β-CD; (**b**) MTX, G-β-CD, MTX/G-β-CD.

The complexes of FA and its structural analog MTX with G-β-CD and Ma-β-CD were studied by analyzing the spectral features of recorded FTIR spectra (Figure 6 and Figure S20). The assignment of IR bands for FA is in accordance with known data [46]. The region in the range of 700 to 950 cm^−1^ belongs to the deformation vibrations of the C–H bonds and also the pulsation vibration of the glucopyranose ring of CD. In this area, the three most intensive bands appear at 765 cm^−1^, 835 cm^−1^, and 879 cm^−1^ for FA and at 762 cm^−1^, 826 cm^−1^, and 918 cm^−1^ for MTX molecules. These bands are also observed as broader and in slightly different positions for both pure G-β-CD and Ma-β-CD. After the complex formation for FA/G-β-CD, the first band (765 cm^−1^) is positioned at the same wavenumber as the corresponding band in spectra of FA, the second band (835 cm^−1^), as well as the third band (879 cm^−1^) and the new band (at 918 cm^−1^) are observed for FA. Those bands are very broad and shifted, or they do not appear for G-β-CD but appear for the complexes in similar positions as in spectra of cyclodextrins, but with higher intensity. Similar behavior is observed for MTX, G-β-CD, and its complex. The region of 1050 to 1100 cm^−1^ is characteristic of the valence vibrations of the C–O stretching bands in the ether and hydroxyl groups of CDs. In this area, very low intensities of bands are observed for FA and MTX molecules; broad bands are observed for both cyclodextrins, while those bands are strengthened in the spectra of their complexes at 1001 cm^−1^, 1034 cm^−1^, as well as bands at 1080 cm^−1^, 1154 cm^−1^ are shifted and their intensities higher. The range of 1300 to 1650 cm^−1^ is attributed to C=C stretching vibrations of the guest molecule. For FA and MTX, as well as FA/G-β-CD, MTX/G-β-CD molecules, a few bands are observed at 1443, 1510, 1598 cm^−1^ and 1450, 1508, 1598 cm^−1^, respectively. The bands at 1593 cm^−1^ and 1598 cm^−1^ assigned to C=O vibrational modes (see Table S7) are relatively strong. However, there are no changes in their position. What is also important is that the band observed at 1334 cm^−1^ shows a decrease in intensity in the IR spectra of FA and MTX cyclodextrins complexes. Additionally, the region 2000–4000 cm^−1^ is presented in Figure S20 (Supporting Information), and it shows a broad band for FA, with the maximum intensity at 3348 cm^−1^ that corresponds to OH groups (see Figure S20a). However, these broad bands change in the spectra of FA/G-β-CD, indicating the interaction between the host/guest molecules. The IR spectra of FA/G-β-CD contain the additional intense bands at 2899 cm^−1^, 2931 cm^−1^, and 2982 cm^−1^ due to C – H asymmetric/symmetric stretching. Similarly, it is observed in the case of MTX and MTX/G-β-CD samples (Figure S20b); however, herein, these relations and intensities are less intense. Similar results are obtained for the complexes of FA and MTX with Ma-β-CD (Figures S21 and S22). Briefly, bands at 765, 835, 1001, 1023, 1334, 1443, 1510, 1598 cm^−1^ and 762, 826, 1001, 1024, 1450, 1508, 1593 cm^−1^ show shifting and bands intensity changes due to FA/M- β -CD, MTX/Ma-β-CD complex formation, respectively. Such behavior of the characteristic features of the recorded IR spectra indicates host/guest interactions between FA or MTX and the host G-β-CD, as well as the Ma-β-CD molecules. All discussed bands are gathered and assigned in Table S7 (Supporting Information).

To conclude, the recorded data for FA and MTX, G-β-CD and FA/G-β-CD, and MTX/G-β-CD (Figure 6) prove that some of the bands observed in cyclodextrin complexes are characteristic for pure FA, MTX, and G-β-CD, while observed changes of these bands are due to the cyclodextrin complexes formation. Similarly, for FA and MTX with Ma-β-CD, changes in position and intensities of respective IR bands observed in the spectra of cyclodextrin complexes are strictly connected with complex formation (Figure S20).

## 4. Conclusions

We obtained four new host/guest complexes based on carbohydrate-derived cyclodextrins (G-β-CD and Ma-β-CD) with FA and its structural analog, anticancer MTX, as guest molecules. All the complexes were obtained as 1:1 adducts, which UV-vis titrations in PBS proved. In all the cases, obtained complexes (FA/G-β-CD, FA/Ma-β-CD, MTX/G-β-CD, MTX/Ma-β-CD) exhibit similar geometry, where the phenyl ring of the guest is fully encapsulated inside the host cavity, with the bicyclic part of the structure interacting with the secondary side of cyclodextrin. At the same time, glutamic acid moiety reaches out from the primary side of the macrocyclic host, as proved by ^1^H NMR and molecular modeling. The glucosyl- and maltosyl-substituents, located on the narrower rim of the cyclodextrin host, take part in the binding of guests, which is illustrated by the geometry obtained from molecular modeling and the increased (doubled) values of stability constants, as compared to the similar complexes formed by unsubstituted β-cyclodextrin. The stability constants obtained for the complexes with Ma-β-CD are of the same order of magnitude as those obtained for the complexes with G-β-CD, which means that the additional glucose unit present in the maltosyl chain of M-β-CD does not interact significantly with the guest molecules (FA and MTX), and therefore, does not interfere in the complexation process (as also seen from ^1^H NMR and molecular modeling). This confirms the results obtained previously for various branched cyclodextrins [47]. To sum up, our studies proved that incorporating the glucose subunit into the structure of the β-cyclodextrin host helps the efficient binding of folate-type guests, such as FA and MTX, and increases the value of stability constants of the host/guest complexes. This result provides a starting point for further research on the complexation of drug molecules with modified CDs, which creates the potential for the targeted delivery of chemotherapeutics and lower doses in treatment to reduce the harmful side effects.

## Figures and Tables

**Table 1:** The values of complex association constants (*K_a_*) measured in phosphate-buffered saline (pH = 7.4).

	FA		MTX	
	Stoichiometry (H:G)	*K_a_* [M^−1^]	Stoichiometry (H:G)	*K_a_* [M^−1^]
β-CD	1:1	131 ± 10.5 [16]	1:1	389.7 ± 104.5 [19]
G-β-CD	1:1	264 ± 0.9	1:1	630 ± 5.5
Ma-β-CD	1:1	251 ± 0.8	1:1	637 ± 8.6

**Table 2:** The proton chemical shifts (δ) of the following: (**a**) FA and FA/G-β-CD; (**b**) MTX and MTX/G-β-CD. Only protons for which important changes appear are presented.

**(a) Signal**	**FA**	**FA + G-β-CD**	
	**δ** **/** **ppm**	**δ** **/** **ppm**	**Δ** **δ** **/ppm**
FA#H3	8.65	8.69	+0.04
FA #H4	4.57	ns*	Ns *
FA #H6	6.79	6.84	+0.05
FA #H7	7.70	7.74	+0.04
FA #H10A	2.05	2.07	+0.02
FA #H10B	2.17	2.22	+0.05
FA #H11	2.25	2.32	+0.07
**(b) Signal**	**MTX**	**MTX + G-β-CD**	
	**δ** **/** **ppm**	**δ** **/** **ppm**	**Δ** **δ** **/ppm**
MTX#H3	8.67	8.50	−0.17
MTX #H4	4.86	4.96	−0.1
MTX #H5	3.24	3.33	−0.07
MTX #H6	6.99	6.68	−0.31
MTX #H7	7.79	7.72	−0.07
MTX #H9	4.36	4.42	−0.06

* not resolved signal.

## Data Availability

Data are contained within the article.

## Supplementary Materials

The following supporting information can be downloaded at https://www.mdpi.com/article/10.3390/pharmaceutics16091161/s1, Figure S1: Experimental titration spectra at pH = 7.4 (PBS) for FA/Ma-β-CD; Figure S2: Experimental titration spectra at pH = 7.4 (PBS) for MTX/Ma-β-CD; Figure S3: Observed absorbance and fitted theoretical curve for the titration of FA (A) with G-β-CD (B) at a wavelength of 281 nm; Figure S4: Observed absorbance and fitted theoretical curve for the titration of FA (A) with Ma-β-CD (B) at a wavelength of 283 nm. Figure S5: Observed absorbance and fitted theoretical curve for the titration of MTX (A) with G-β-CD (B) at a wavelength of 300 nm; Figure S6. Observed absorbance and fitted theoretical curve for the titration of MTX (A) with Ma-β-CD (B) at a wavelength of 281 nm; Figure S7: Stacked ^1^H NMR spectra in D_2_O of (from top to bottom): FA, Ma-β-CD, FA/M-β-CD; Figure S8: Stacked ^1^H NMR spectra in D_2_O of (from top to bottom): MTX, Ma-β-CD, MTX/G- β-CD; Figure S9: 1:1 complexes of (H:G) FA/G-β-CD: (a) Capped-sticks mode; (b) Space-filling mode; Figure S10: 1:1 complexes of (H:G) FA/Ma-β-CD: (a) Capped-sticks mode; (b) Space-filling mode; Figure S11: Conformation of FA in its inclusion complexes with the following: (a) G-β-CD; (b) M-β-CD; Figure S12: 1:1 complexes of (H:G) MTX/G-β-CD: (a) Host and guest in space-filling mode; (b) Guest molecule in space-filling mode; Figure S13: 1:1 complexes of (H:G) MTX/Ma-β-CD: (a) Host and guest in space-filling mode; (b) Guest molecule in space-filling mode; Figure S14: Conformation of MTX in its inclusion complexes with the following: (a) G-β-CD; (b) M-β-CD; Figure S15: 1:1 complexes of (H:G) FA/G-β-CD (second conformation); Figure S16: 1:1 complexes of (H:G) FA/Ma-β-CD (second conformation); Figure S17: 1:1 complexes of (H:G) MTX/G-β-CD (second conformation); Figure S18: 1:1 complexes of (H:G) MTX/Ma-β-CD (second conformation); Figure S19: DSC (a) and TGA (b) curves of FA, MTX and their solid state complexes with M-β-CD; Figure S20: The FTIR spectra in the region 2000–4000 cm^−1^ recorded for the following: (a) FA, M-β-CD, FA + M-β-CD; (b) MTX, G-β-CD, MTX + M-β-CD; Figure S21: Stacked FTIR spectra: (a) FA, M-β-CD, FA + M-β-CD; (b) MTX, G-β-CD, MTX + M-β-CD; Figure S22: The FTIR spectra in the region 2000–4000 cm^−1^ recorded for the following: (a) FA, M-β-CD, FA + M-β-CD; (b) MTX, G-β-CD, MTX + M-β-CD; Table S1: The proton chemical shifts (δ) of FA and M-β-CD/FA. Table S2: The proton chemical shifts (δ) MTX and Ma-β-CD/MTX; Table S3: Cartesian coordinates of obtained structures of FA complexes (conformation 1); Table S4: Cartesian coordinates of obtained structures of FA complexes (conformation 2); Table S5: Cartesian coordinates of obtained structures of MTX complexes (conformation 1); Table S6: Cartesian coordinates of obtained structures of MTX complexes (conformation 2); Table S7: The FTIR wavenumbers assignment in sequence: FA, MTX, G-β-CD, FA + G-β-CD, MTX + G-β-CD, and M-β-CD, FA + M-β-CD, MTX + M-β-CD.
