# Supplementary material for: Synergetic Effect of β-Cyclodextrin and Its Simple Carbohydrate Substituents on Complexation of Folic Acid and Its Structural Analog Methotrexate
Source: Pharmaceutics. 2024 Sep 3;16(9):1161. doi: 10.3390/pharmaceutics16091161 (PMC11435387; doi:10.3390/pharmaceutics16091161)
Supplement: Supplementary file 1 [file pharmaceutics-16-01161-s001.zip › pharmaceutics-3077498-supplementary.pdf]

SUPPORTING INFORMATION

Synergetic effect of β-cyclodextrin and its simple carbohydrates substituents on the complexation of folic acid and its structural analogue methotrexate

Magdalena Ceborska,[ab] Karolina Kędra,[b] Aleksandra Siklitckaia,[b] Aneta Aniela Kowalska[b]

[a] *Faculty of Mathematics and Natural Sciences, Cardinal Stefan Wyszyński University Wóycickiego 1/3, 01-938 Warsaw, Poland*

[b] *Institute of Physical Chemistry, Polish Academy of Sciences, Kasprzaka 44/52, 01-224 Warsaw, Poland*

Contents:

1. UV-Vis……………………………………………………………………..S2

2. ^{1}H NMR ………………………………………………………..……….……S5

3. Molecular modeling …………………………………………………..……….S7

4. DSC/TG ....……………………………………………………….……..S33

5. FTIR……………………….………………………………………………….S34

1. UV-Vis.

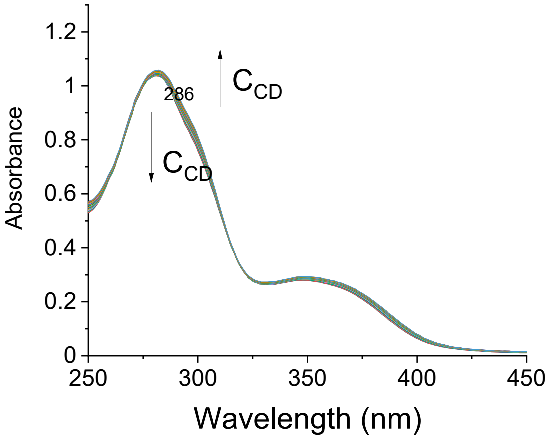

Figure S1. Experimental titration spectra at pH=7.4 (PBS) for FA/Ma-β–CD.

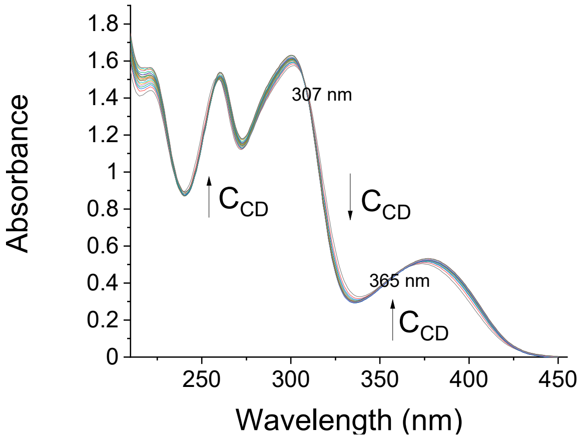

Figure S2. Experimental titration spectra at pH=7.4 (PBS) for MTX/Ma-β–CD.

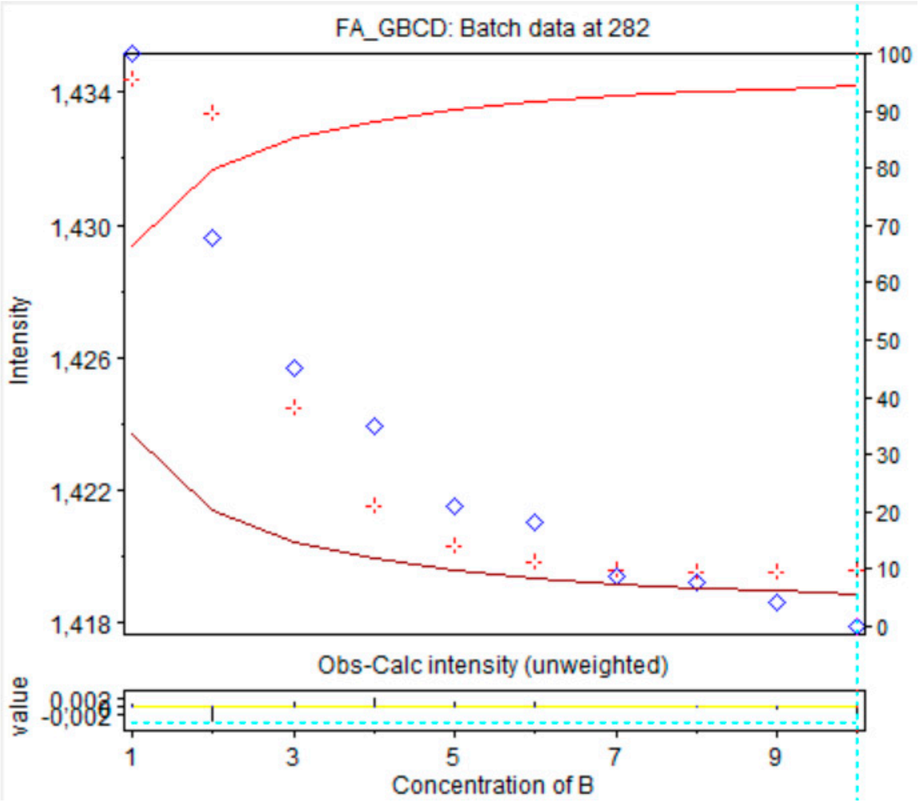

Figure S3. Observed absorbance and fitted theoretical curve for the titration of FA (A) with G-β-CD (B) at a wavelength of 281 nm. Figure description: Intensity = Absorbance, "◇ " – the experimental data (titration points), "+" – fitted theoretical points, " —"- % of AB (meaning the appropriate 1:1 inclusion complex), " —" - % of A (meaning the guest molecule).

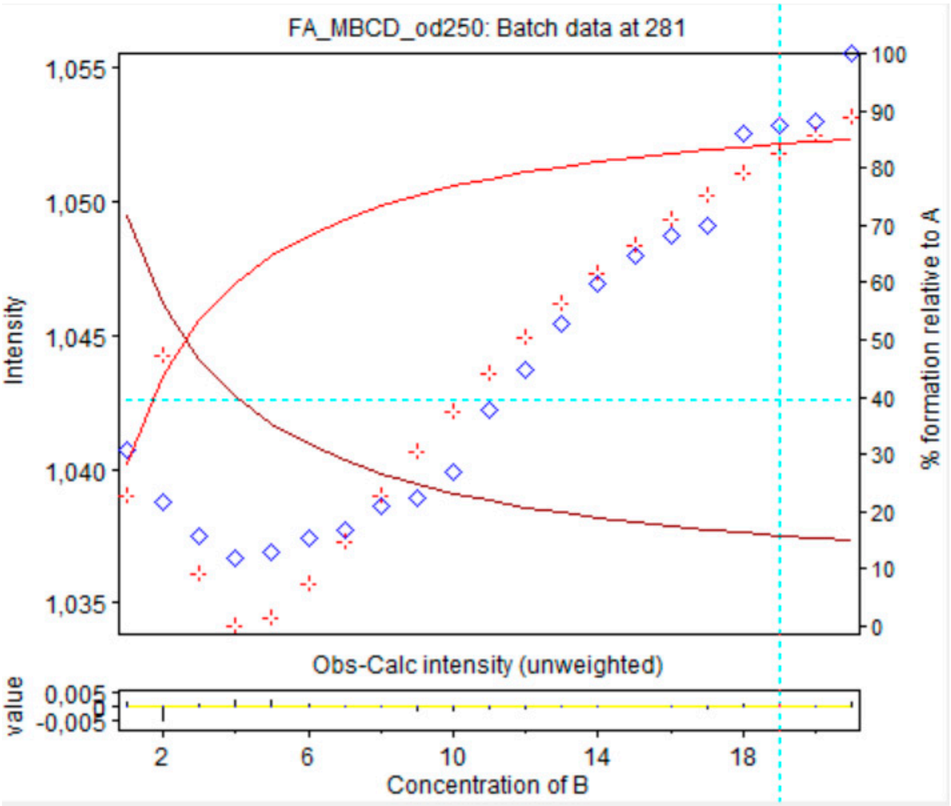

Figure S4. Observed absorbance and fitted theoretical curve for the titration of FA (A) with Ma-β-CD (B) at a wavelength of 283 nm. Figure description: Intensity = Absorbance, "◇ " – the experimental data (titration points), "+" – fitted theoretical points, " —"- % of AB (meaning the appropriate 1:1 inclusion complex), " —" - % of A (meaning the guest molecule).

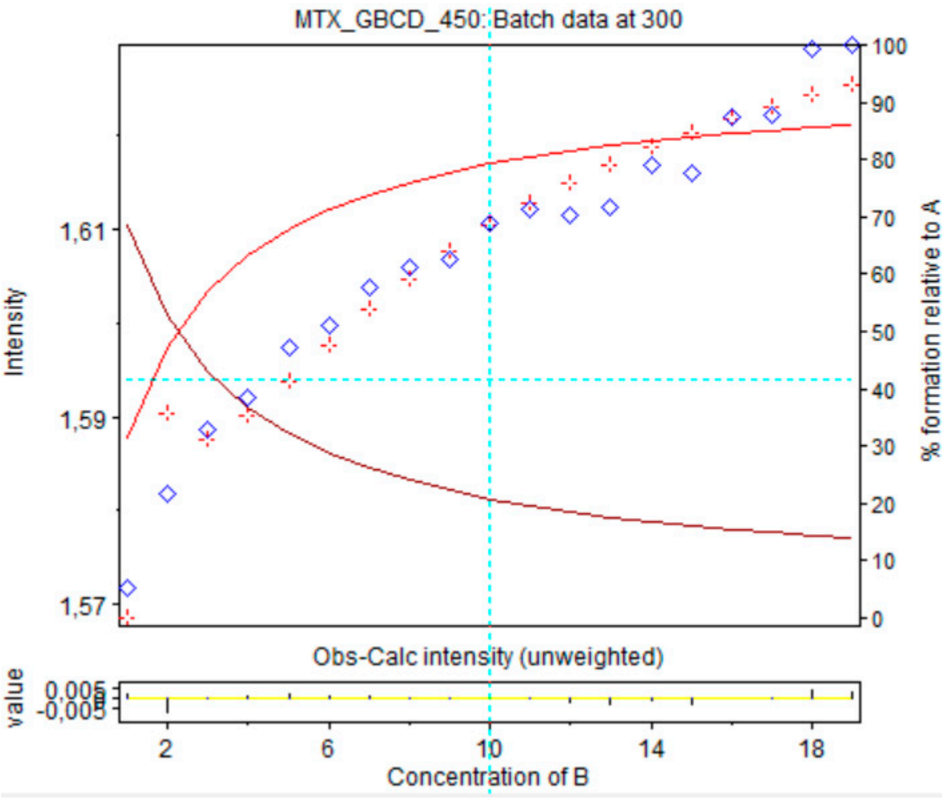

Figure S5. Observed absorbance and fitted theoretical curve for the titration of MTX (A) with G-β-CD (B) at a wavelength of 300 nm. Figure description: Intensity = Absorbance, ”◇ ” – the experimental data (titration points), ”+” – fitted theoretical points, ” —”- % of AB (meaning the appropriate 1:1 inclusion complex), ” —” - % of A (meaning the guest molecule).

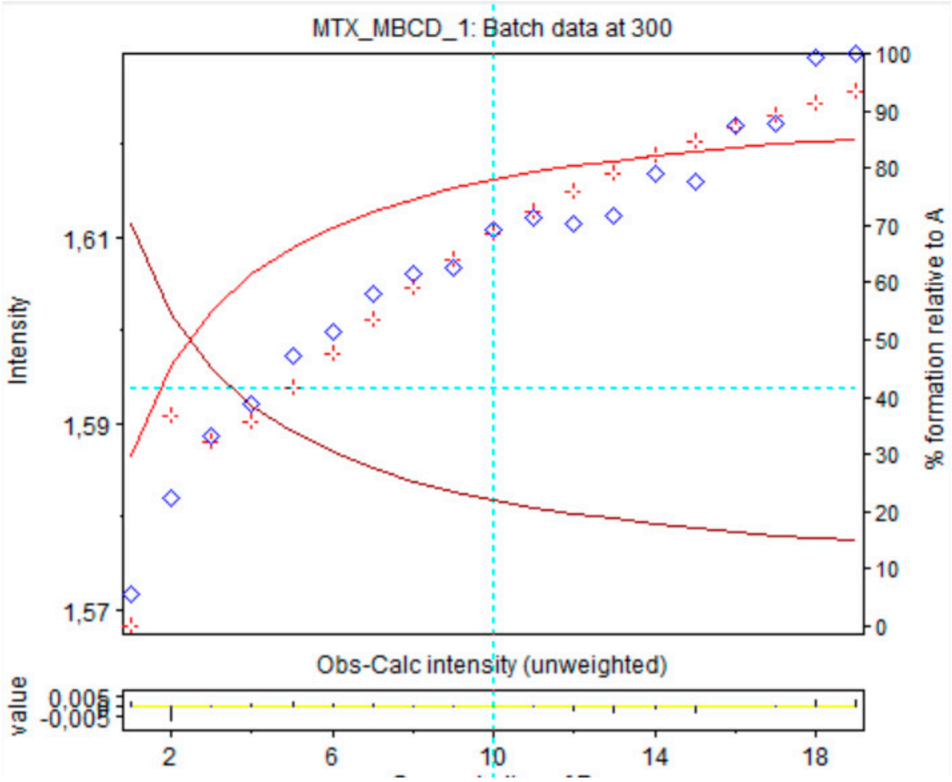

Figure S6. Observed absorbance and fitted theoretical curve for the titration of MTX (A) with Ma-β-CD (B) at a wavelength of 281 nm. Figure description: Intensity = Absorbance, "◇ " – the experimental data (titration points), "+" – fitted theoretical points, " —"- % of AB (meaning the appropriate 1:1 inclusion complex), " —" - % of A (meaning the guest molecule).

2. NMR

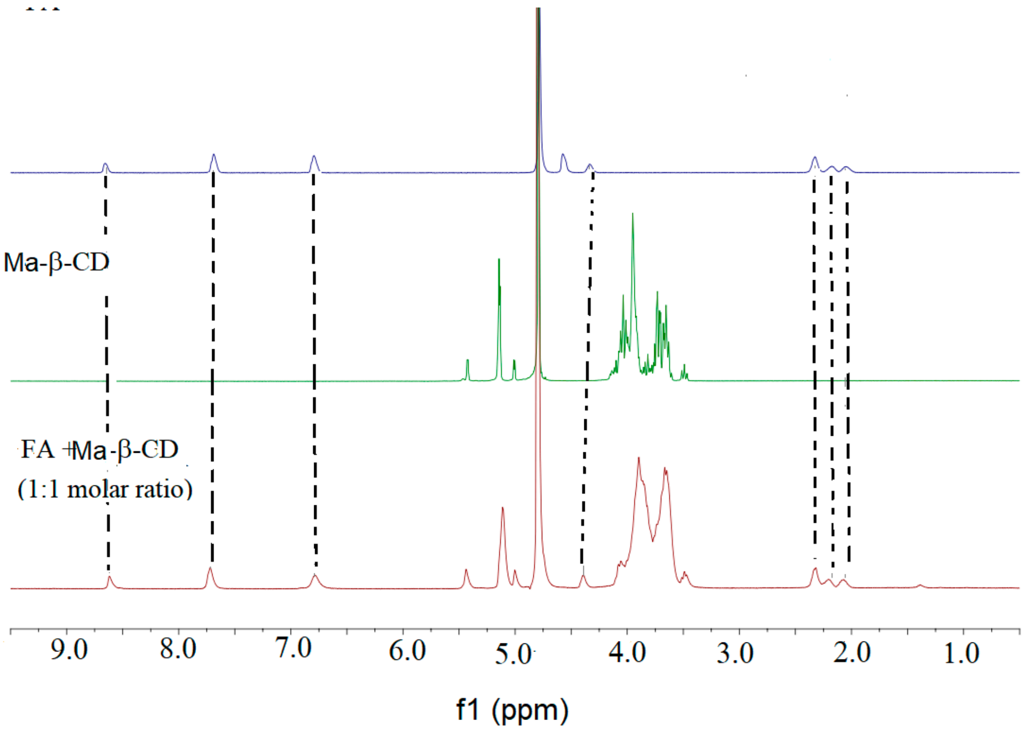

Figure S7. Stacked ^{1}H NMR spectra in D_2O of FA, Ma-β–CD, FA/Ma-β–CD, (from top to bottom).

Table S1. The proton chemical shifts (δ) of FA and Ma-β–CD/FA. Only protons for which important changes appear are presented.

Signal	FA	FA/Ma-β–CD	
	δ /ppm	δ /ppm	Δδ /ppm
FA#H3	8.65	8.60	-0.05
FA #H4	4.57	ns*	ns
FA #H6	6.79	6.71	-0.08
FA #H7	7.69	7.71	+0.02
FA #H10A	2.05	2.05	–
FA #H10B	2.17	2.19	+0.02
FA #H11	2.25	2.30	+0.05

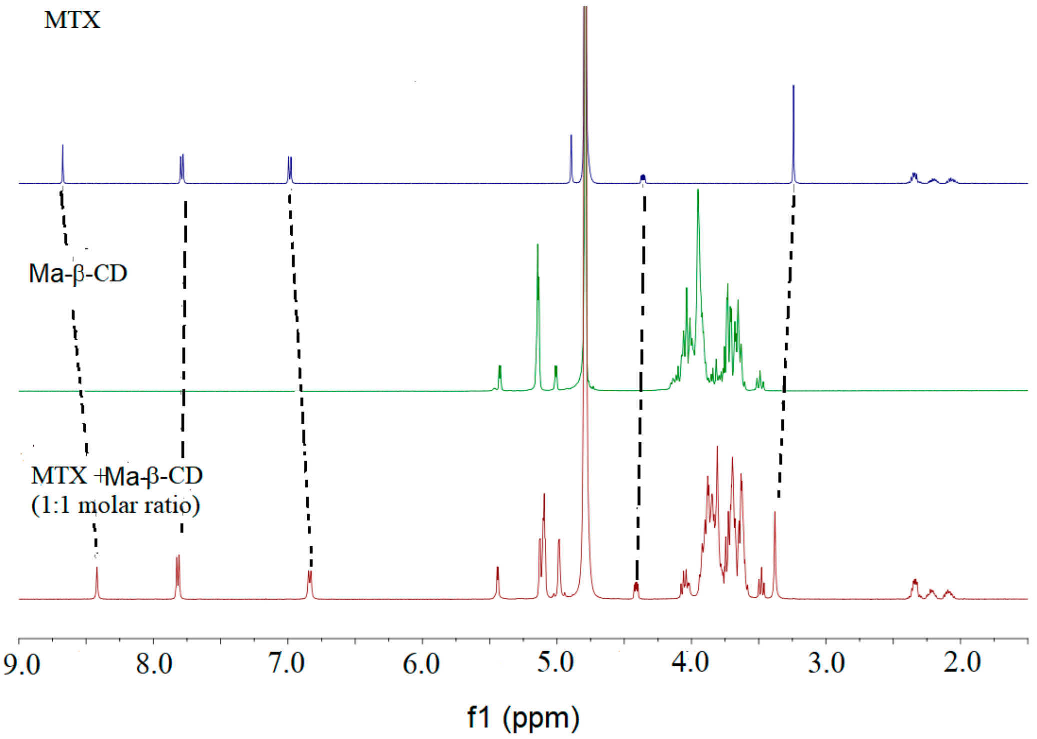

Figure S8. Stacked ^{1}H NMR spectra in D_2O of MTX, Ma-β–CD, MTX/Ma-β–CD; (from top to bottom).

Table S2. The proton chemical shifts (δ) of MTX and M-β–CD/MTX. Only protons for which important changes appear are presented.

Signal	MTX	MTX/Ma-β–CD	
	δ /ppm	δ /ppm	Δδ /ppm
MTX#H3	8.67	8.42	-0.25
MTX #H4	4.86	4.99	+0.13
MTX #H5	3.24	3.38	+0.14
MTX #H6	6.99	6.80	-0.19
MTX #H7	7.79	7.82	+0.03
MTX #H9	4.36	4.41	+0.05

* not resolved signal

3. Molecular modeling

a) **b)**

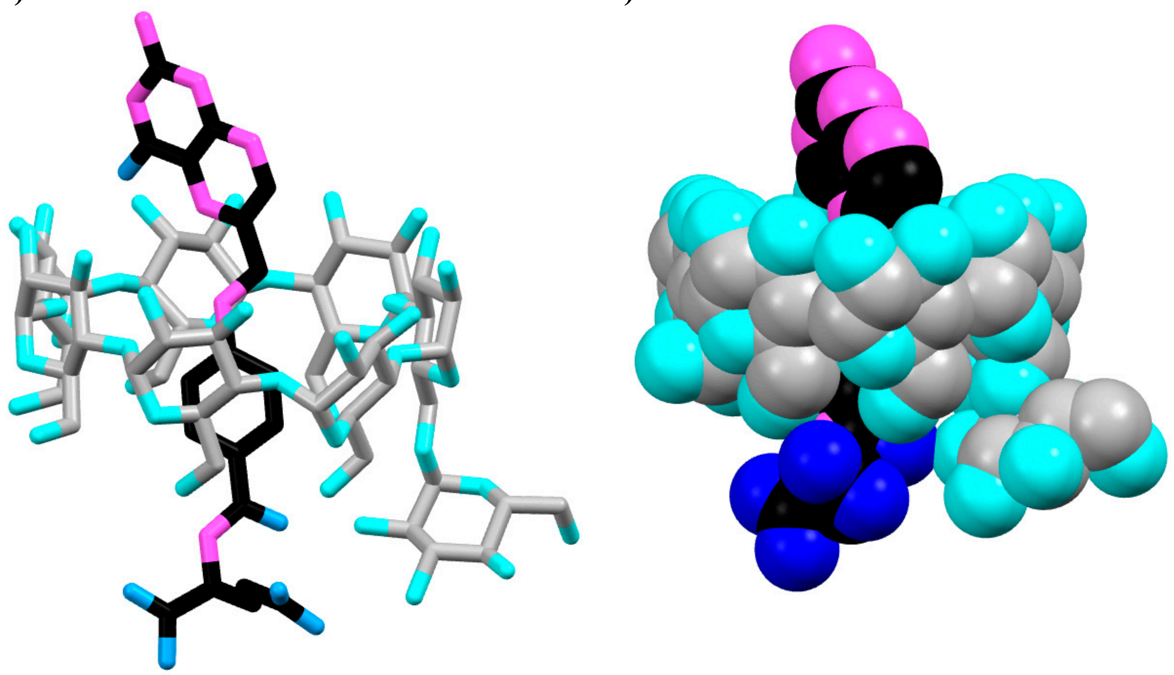

Figure S9. 1:1 complexes of (H:G) FA/G-β–CD (first conformation) a) capped-sticks mode b) space-filling mode.

a) **b)**

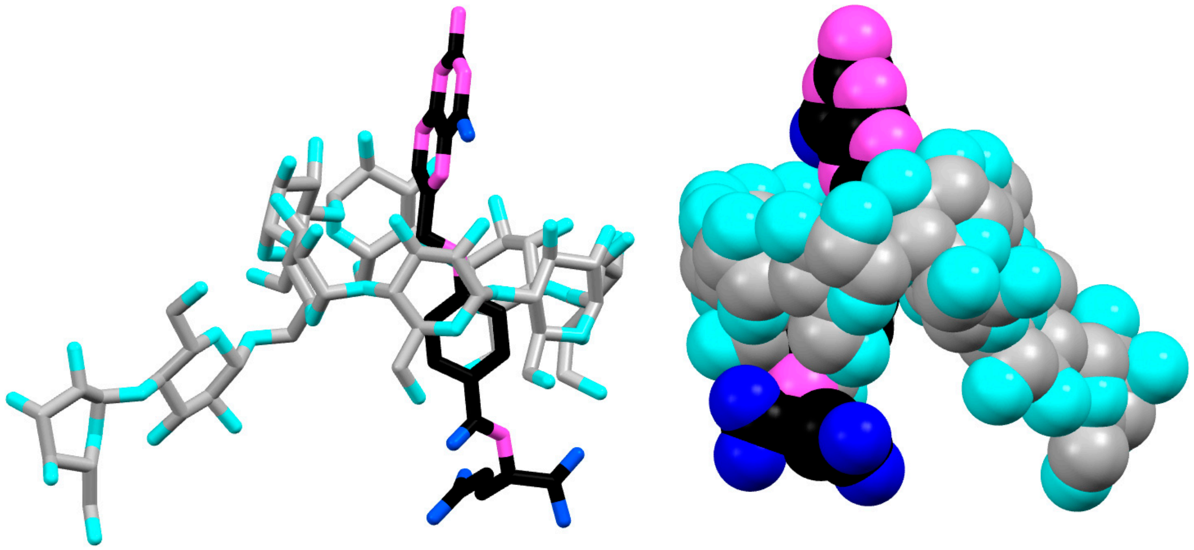

Figure S10. 1:1 complexes of (H:G) FA/Ma-β–CD (first conformation) a) capped-sticks mode b) space-filling mode.

a)

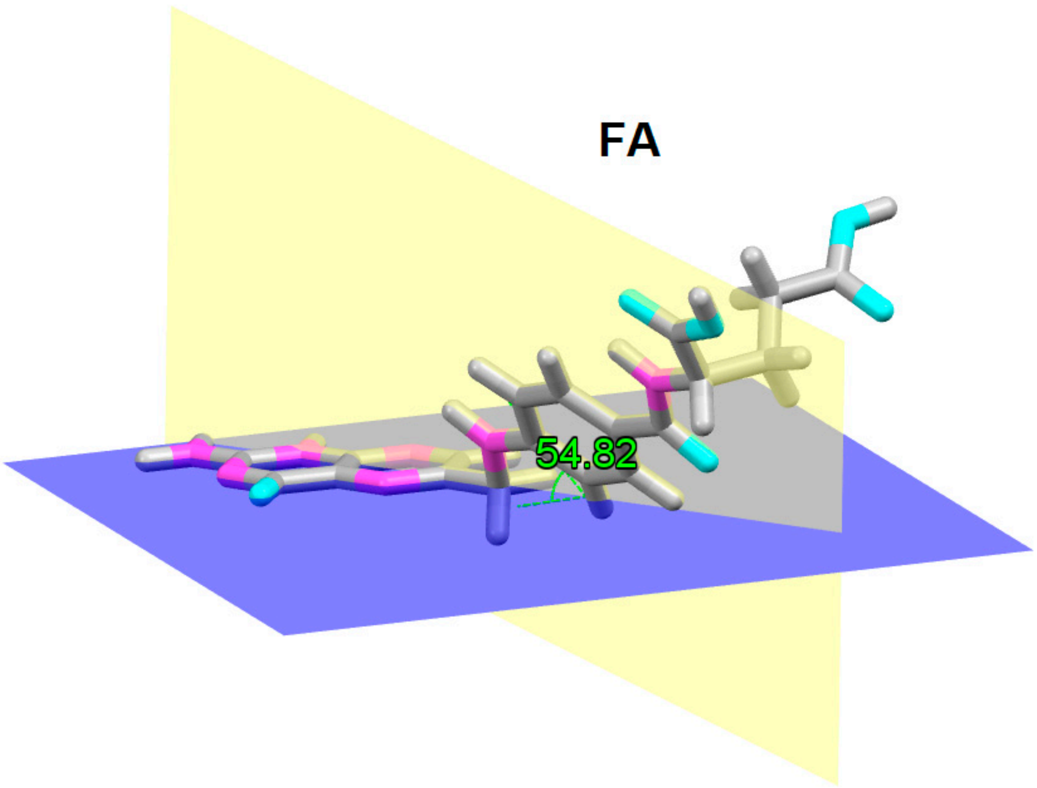

b)

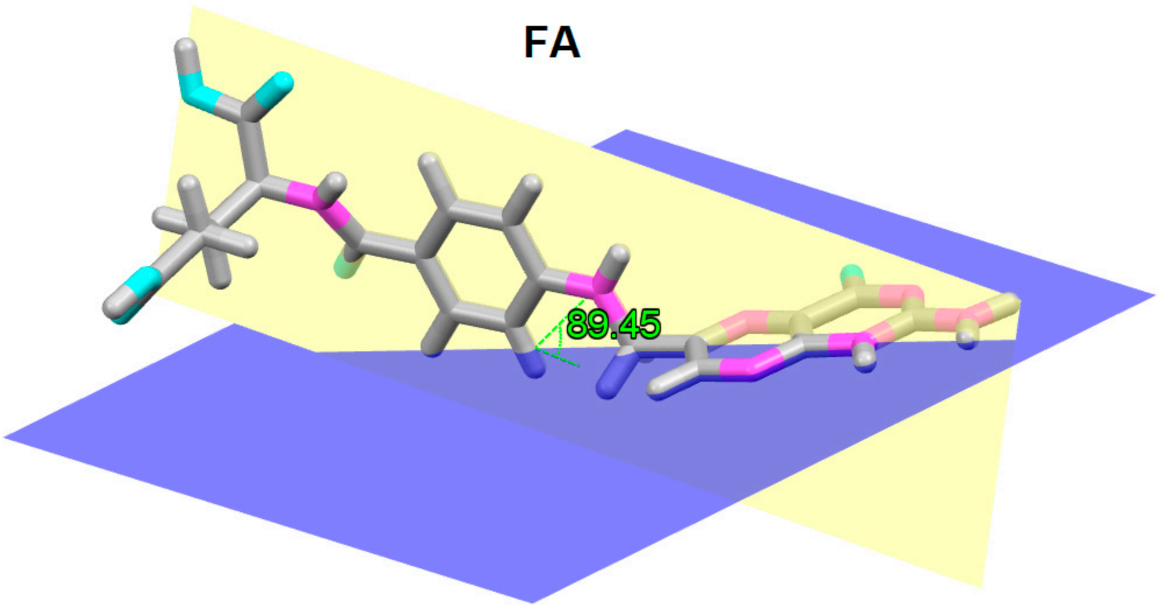

Figure S11. Conformation of FA in its inclusion complexes with a) G-β–CD b) Ma-β–CD.

a) **b)**

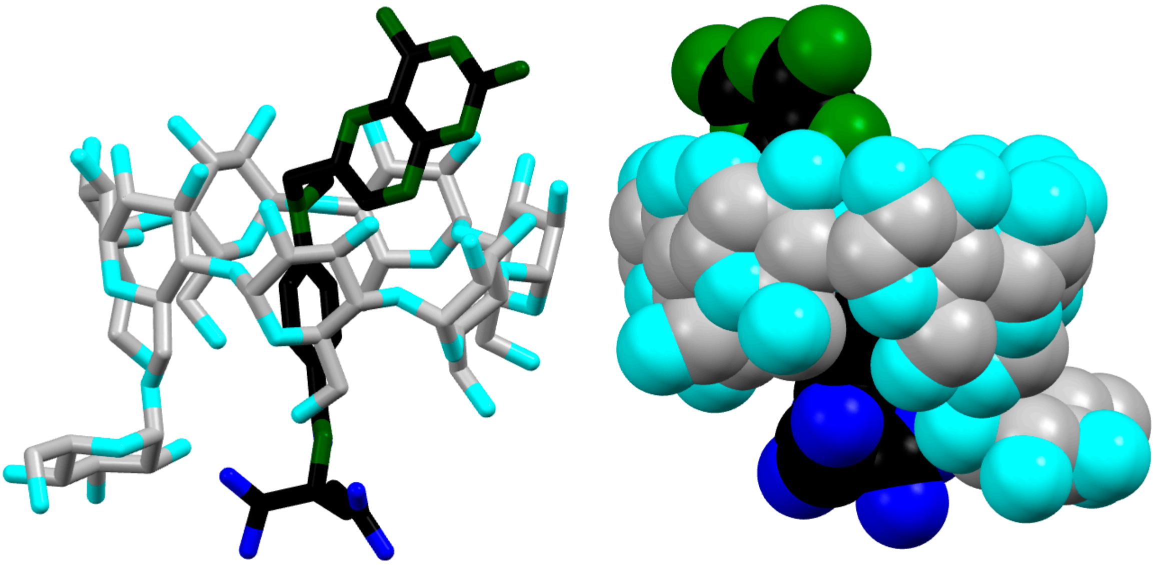

Figure S12. 1:1 complexes of (H:G) MTX/G-β–CD (first conformation) a) host and guest in space-filling mode b) guest molecule in space-filling mode.

a) **b)**

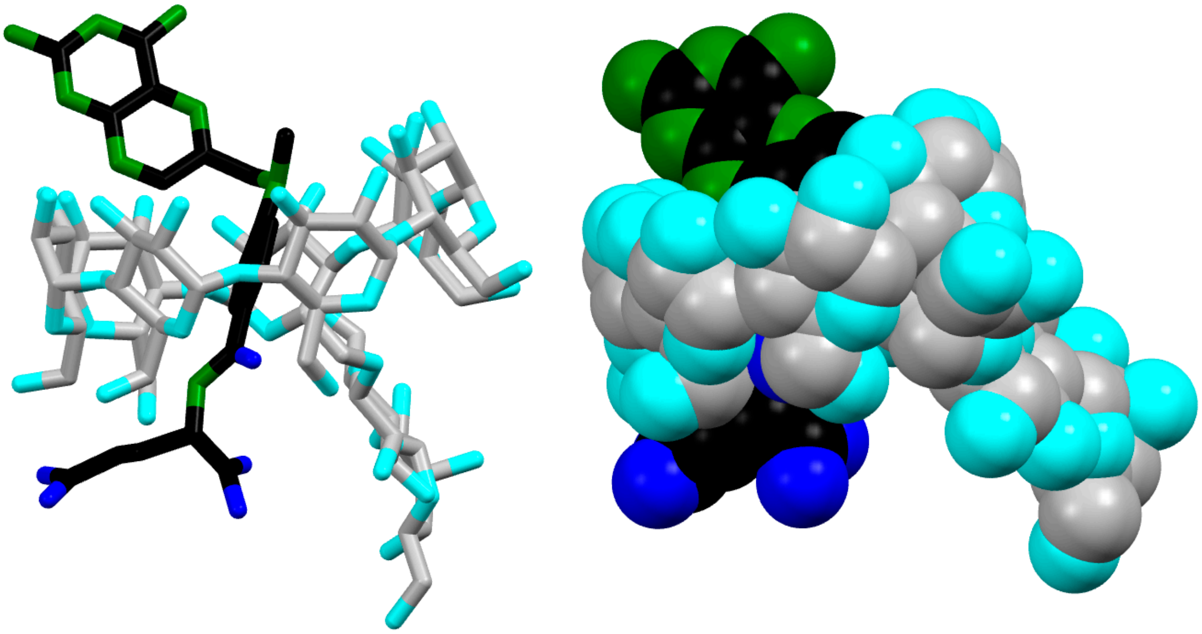

Figure S13. 1:1 complexes of (H:G) MTX/Ma-β–CD (first conformation) a) host and guest in space-filling mode b) guest molecule in space-filling mode.

a)

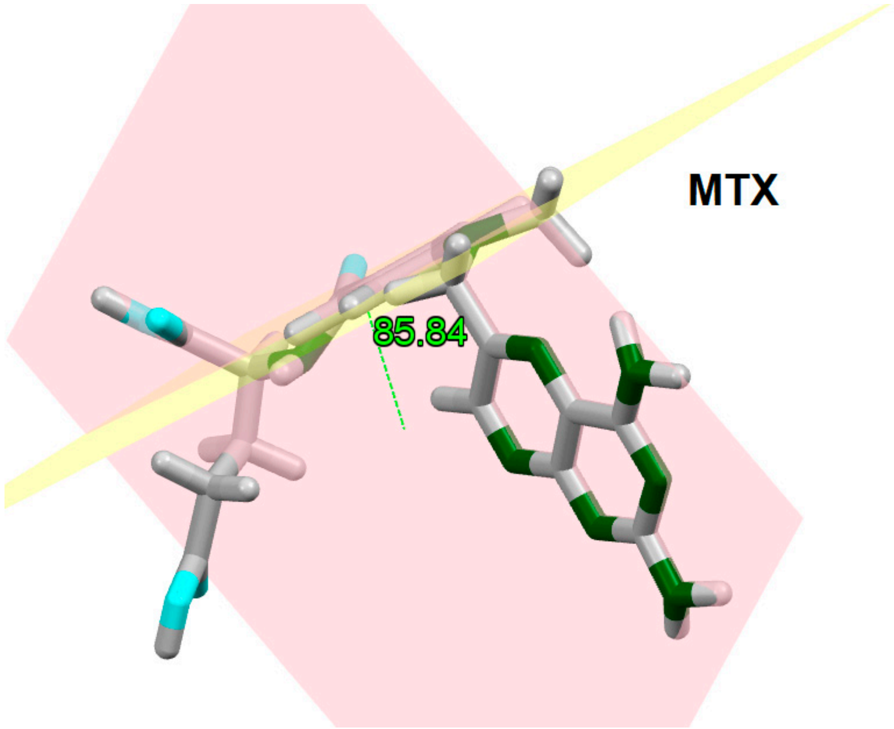

b)

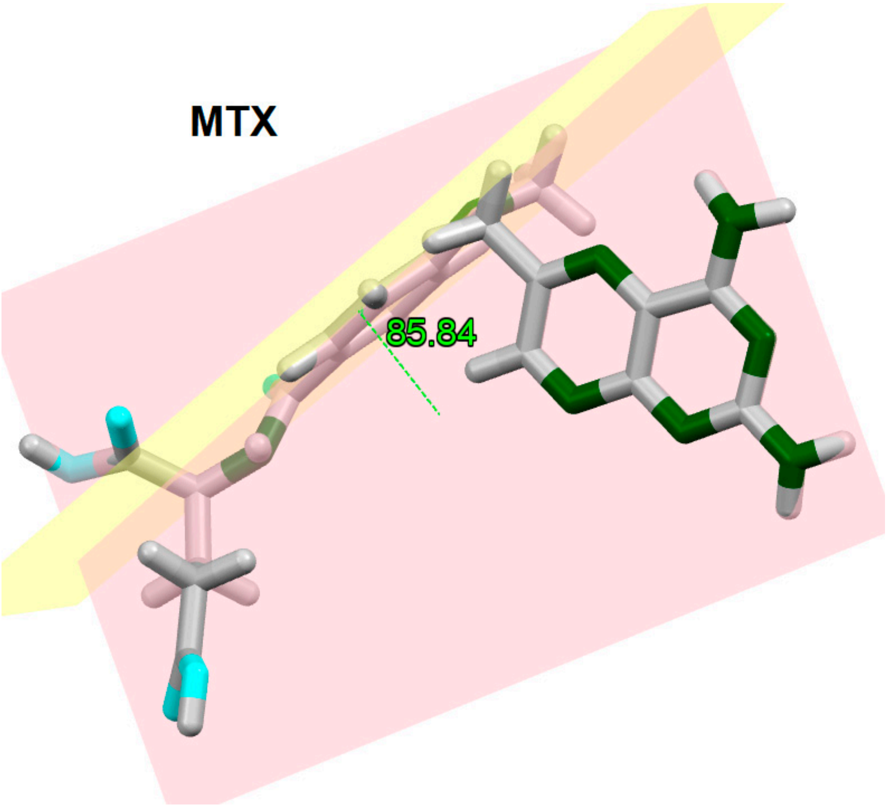

Figure S14. Conformation of MTX in its inclusion complexes with a) G-β–CD, b) Ma-β–CD.

a) **b)**

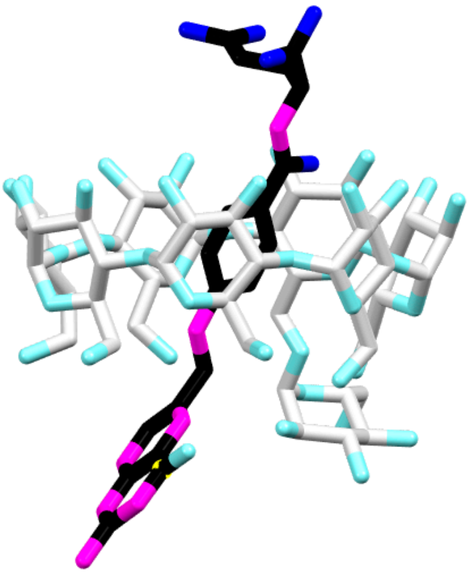

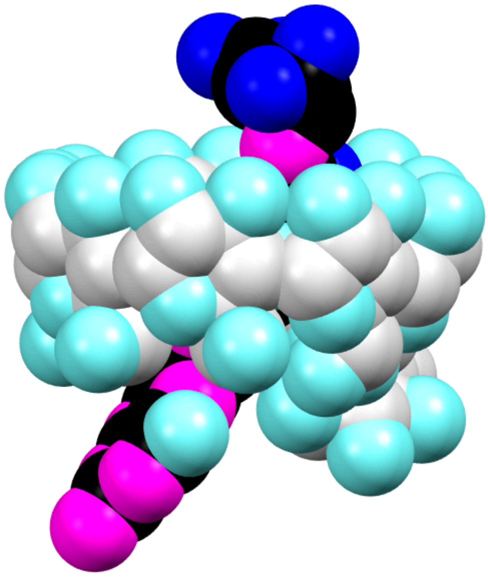

Figure S15. 1:1 complexes of (H:G) FA/G-β–CD (second conformation); a) capped-sticks mode; b) space-filling mode.

a) b)

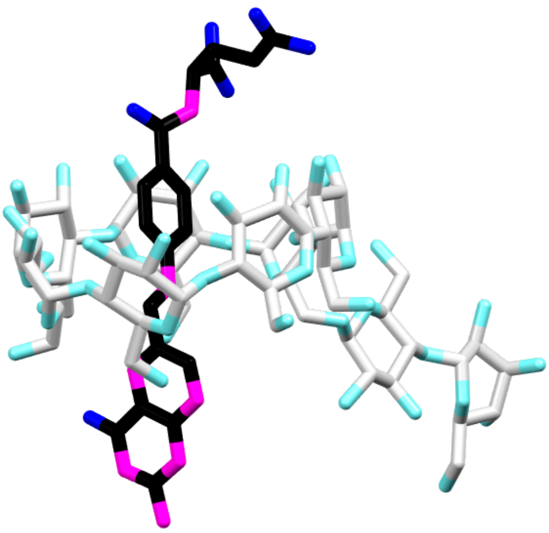

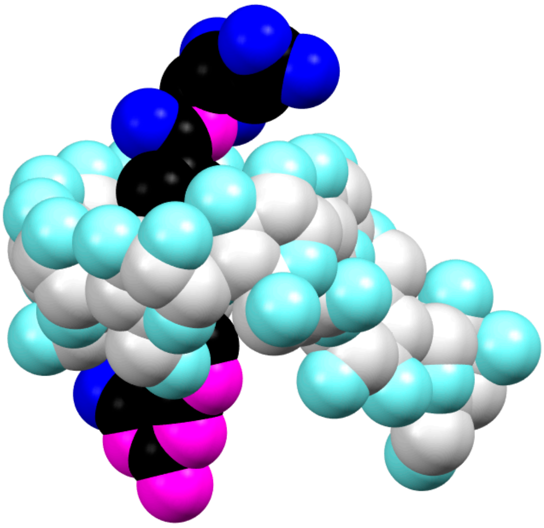

Figure S16. 1:1 complexes of (H:G) FA/Ma-β–CD (second conformation) a) capped-sticks mode b) space-filling mode.

a)

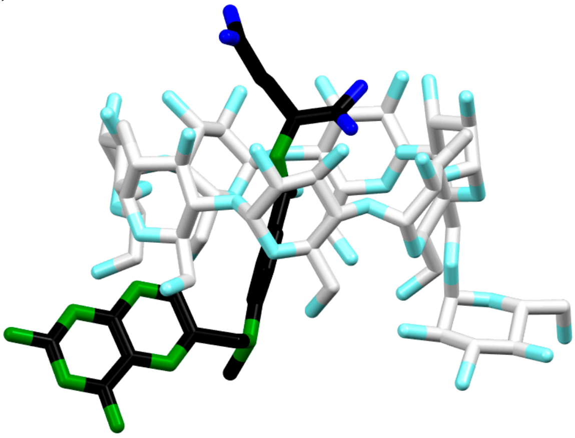

b)

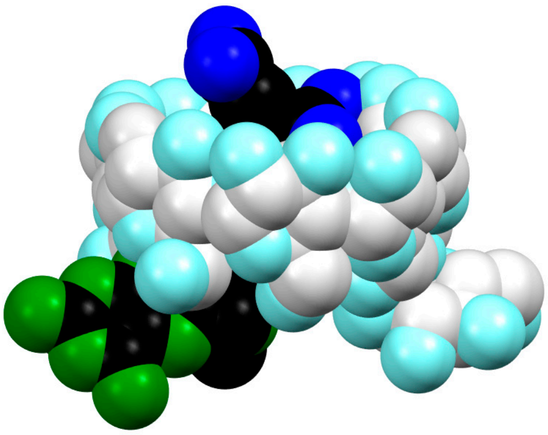

Figure S17. 1:1 complexes of (H:G) MTX/G-β–CD (second conformation); a) capped-sticks mode; b) space-filling mode.

a)

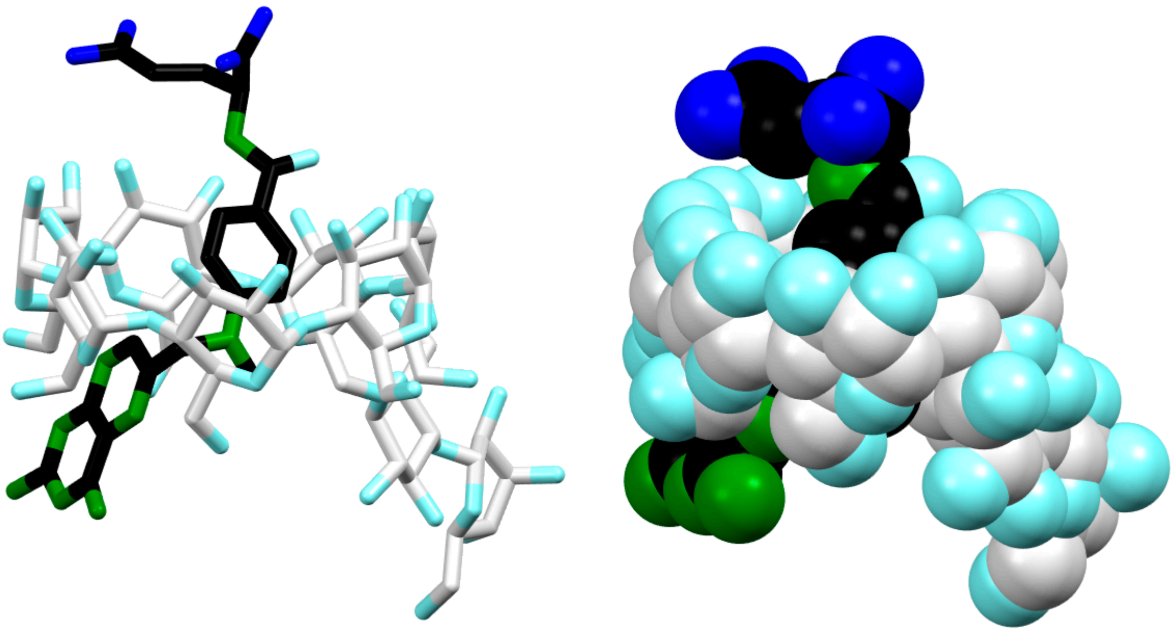

Figure S18. 1:1 complexes of (H:G) MTX/Ma-β–CD (second conformation); a) capped-sticks mode; b) space-filling mode.

Table S3. Cartesian coordinates of obtained structures of FA complexes (conformation 1).

FA/ G-β–CD				FA/ Ma-β–CD			
**Atom**	**x**	**y**	**z**	**Atom**	**x**	**y**	**z**
H	3.388000	5.972000	-10.526000	C	-0.944000	5.954000	1.307000
H	1.243000	6.283000	-10.886000	C	-0.429000	5.614000	2.735000
H	2.398000	2.935000	-10.598000	C	0.222000	4.207000	2.765000
H	4.908000	0.933000	-10.618000	C	1.256000	4.058000	1.609000
O	3.295000	3.328000	-10.432000	C	0.655000	4.505000	0.246000
O	1.397000	5.451000	-10.367000	C	1.762000	4.654000	-0.819000
C	2.665000	5.568000	-9.778000	C	-5.850000	5.121000	-0.544000
H	5.269000	4.715000	-9.375000	C	-5.682000	5.548000	0.943000
O	4.598000	0.775000	-9.689000	C	-4.288000	5.123000	1.469000
H	6.256000	2.845000	-9.639000	C	-3.195000	5.669000	0.503000
H	2.313000	7.499000	-8.965000	C	-3.481000	5.216000	-0.952000
C	3.168000	4.187000	-9.329000	C	-2.532000	5.833000	-2.006000
H	6.609000	0.458000	-9.317000	C	-8.062000	0.615000	-2.276000
C	2.613000	6.508000	-8.557000	C	-8.772000	1.472000	-1.185000
C	4.531000	4.335000	-8.631000	C	-7.729000	2.297000	-0.389000
C	5.716000	0.943000	-8.856000	C	-6.877000	3.119000	-1.398000

O	3.917000	6.640000	-8.013000	C	-6.240000	2.177000	-2.452000
C	6.005000	2.442000	-8.632000	C	-5.479000	2.914000	-3.580000
O	-0.974000	6.438000	-8.490000	C	-6.448000	-4.422000	-1.652000
H	-1.784000	5.868000	-8.556000	C	-7.823000	-3.982000	-1.067000
H	2.455000	3.753000	-8.587000	C	-7.842000	-2.457000	-0.797000
O	6.675000	6.185000	-7.774000	C	-7.398000	-1.692000	-2.079000
H	4.313000	-1.246000	-7.908000	C	-6.044000	-2.240000	-2.599000
O	4.873000	3.082000	-8.051000	C	-5.717000	-1.684000	-4.004000
H	7.518000	6.348000	-7.276000	C	-2.501000	-5.881000	1.585000
C	4.410000	5.402000	-7.509000	C	-3.890000	-5.741000	2.271000
O	1.699000	6.012000	-7.580000	C	-4.810000	-4.818000	1.434000
H	0.880000	7.989000	-7.503000	C	-4.844000	-5.291000	-0.049000
O	7.149000	2.586000	-7.801000	C	-3.427000	-5.589000	-0.618000
O	5.266000	-1.111000	-7.667000	C	-3.506000	-6.415000	-1.924000
C	5.762000	5.680000	-6.838000	C	1.791000	-2.871000	2.448000
C	5.456000	0.270000	-7.501000	C	0.906000	-3.320000	3.656000
H	6.161000	4.753000	-6.387000	C	-0.547000	-3.624000	3.209000
C	0.837000	6.992000	-7.008000	C	-0.531000	-4.548000	1.955000
C	-0.612000	6.480000	-7.134000	C	0.325000	-3.890000	0.843000
H	3.733000	5.017000	-6.713000	C	0.459000	-4.731000	-0.448000
H	-1.551000	8.402000	-6.840000	C	2.878000	2.252000	1.597000
H	4.549000	0.726000	-7.032000	C	3.231000	1.567000	2.950000
H	7.531000	0.004000	-6.998000	C	2.338000	0.324000	3.168000
H	-0.651000	5.454000	-6.696000	C	2.472000	-0.618000	1.937000
C	6.645000	0.516000	-6.558000	C	2.253000	0.141000	0.601000
C	-1.570000	7.398000	-6.355000	C	2.703000	-0.693000	-0.621000
H	5.612000	6.428000	-6.028000	O	-1.535000	5.725000	3.610000
H	3.325000	7.076000	-5.642000	O	0.825000	4.093000	4.036000
C	6.926000	2.041000	-6.503000	O	1.528000	2.660000	1.517000
O	-2.872000	6.874000	-6.395000	O	0.050000	5.802000	0.332000
O	9.290000	1.795000	-6.117000	O	1.143000	4.603000	-2.082000
H	6.760000	-3.171000	-5.851000	O	-6.750000	4.965000	1.663000
H	-3.484000	7.608000	-6.126000	O	-4.165000	5.665000	2.766000
H	6.057000	2.531000	-6.034000	O	-1.965000	5.066000	0.905000
O	2.678000	9.018000	-5.897000	O	-4.805000	5.574000	-1.362000
C	1.163000	7.188000	-5.500000	O	-2.416000	7.225000	-1.943000
H	3.611000	9.319000	-5.739000	O	-9.521000	0.588000	-0.374000
H	10.015000	2.039000	-5.485000	O	-8.457000	3.134000	0.484000
H	8.248000	3.499000	-5.608000	O	-5.804000	3.714000	-0.670000
C	2.559000	7.773000	-5.264000	O	-7.233000	1.379000	-3.107000
C	8.126000	2.393000	-5.619000	O	-6.191000	3.950000	-4.191000
O	5.996000	-2.777000	-5.354000	O	-8.034000	-4.734000	0.112000
H	8.067000	-1.077000	-5.279000	O	-9.169000	-2.142000	-0.436000
H	1.157000	6.201000	-4.982000	O	-7.187000	-0.333000	-1.697000
O	6.267000	0.051000	-5.269000	O	-6.074000	-3.665000	-2.769000
O	0.227000	8.072000	-4.896000	O	-4.402000	-1.963000	-4.400000
C	-1.084000	7.531000	-4.900000	O	-3.654000	-5.248000	3.573000
H	-3.272000	4.662000	-4.760000	O	-6.086000	-4.878000	2.036000
H	2.714000	7.895000	-4.169000	O	-5.364000	-4.182000	-0.786000
H	-1.748000	8.288000	-4.428000	O	-2.608000	-6.353000	0.269000

C	7.207000	-0.805000	-4.628000	O	-4.165000	-7.646000	-1.815000
H	7.934000	2.053000	-4.583000	O	0.997000	-2.362000	4.684000
O	-1.113000	6.268000	-4.234000	O	-1.178000	-4.253000	4.301000
C	6.536000	-2.135000	-4.229000	O	-1.867000	-4.627000	1.458000
O	-3.670000	5.033000	-3.929000	O	1.658000	-3.666000	1.303000
H	7.290000	-2.797000	-3.741000	O	0.845000	-6.060000	-0.259000
H	4.651000	-1.220000	-3.714000	O	3.072000	2.535000	3.968000
O	7.743000	-0.178000	-3.472000	O	2.807000	-0.303000	4.342000
H	-2.031000	4.088000	-3.093000	O	1.418000	-1.580000	2.012000
C	5.423000	-1.854000	-3.210000	O	2.973000	1.376000	0.512000
C	-1.807000	6.263000	-2.992000	O	3.993000	-1.257000	-0.515000
C	-2.674000	4.986000	-2.941000	H	-1.232000	7.030000	1.209000
H	-2.500000	7.126000	-2.857000	H	0.321000	6.389000	3.032000
H	0.700000	7.529000	-2.624000	H	-0.573000	3.409000	2.687000
O	4.855000	-3.067000	-2.790000	H	2.175000	4.654000	1.838000
H	5.984000	0.863000	-3.022000	H	-0.113000	3.764000	-0.094000
H	-4.923000	3.802000	-1.885000	H	2.324000	5.601000	-0.691000
C	6.724000	0.180000	-2.544000	H	2.489000	3.820000	-0.723000
H	3.883000	-2.907000	-2.662000	H	-6.751000	5.579000	-1.017000
H	-0.117000	5.369000	-1.916000	H	-5.792000	6.660000	1.006000
C	6.002000	-1.085000	-2.008000	H	-4.218000	4.007000	1.538000
C	-0.815000	6.226000	-1.796000	H	-3.144000	6.786000	0.576000
C	0.071000	7.471000	-1.718000	H	-3.370000	4.097000	-1.024000
H	-0.570000	8.376000	-1.628000	H	-1.540000	5.342000	-1.928000
O	-4.042000	3.649000	-1.455000	H	-2.922000	5.658000	-3.026000
C	-3.324000	4.851000	-1.554000	H	-8.789000	0.161000	-2.991000
H	-2.492000	1.408000	-1.440000	H	-9.493000	2.159000	-1.694000
H	-4.005000	5.717000	-1.372000	H	-7.074000	1.619000	0.213000
H	6.754000	-1.747000	-1.521000	H	-7.518000	3.899000	-1.884000
O	7.997000	2.124000	-1.931000	H	-5.507000	1.496000	-1.947000
O	4.924000	-0.707000	-1.164000	H	-4.511000	3.278000	-3.176000
C	7.370000	0.982000	-1.412000	H	-5.264000	2.225000	-4.417000
H	8.099000	0.357000	-0.846000	H	-6.481000	-5.463000	-2.051000
H	-0.464000	1.356000	-0.744000	H	-8.618000	-4.250000	-1.808000
O	-2.514000	1.207000	-0.468000	H	-7.159000	-2.211000	0.055000
O	0.913000	7.377000	-0.601000	H	-8.186000	-1.770000	-2.872000
H	8.929000	1.868000	-2.155000	H	-5.223000	-1.972000	-1.887000
O	-1.527000	6.124000	-0.568000	H	-6.440000	-2.046000	-4.761000
H	1.484000	8.189000	-0.615000	H	-5.746000	-0.579000	-3.997000
O	-1.332000	3.796000	-0.615000	H	-1.862000	-6.656000	2.074000
H	2.465000	-1.063000	-0.607000	H	-4.343000	-6.759000	2.362000
C	-2.230000	4.894000	-0.471000	H	-4.450000	-3.760000	1.496000
H	6.599000	1.319000	-0.690000	H	-5.488000	-6.205000	-0.146000
C	-1.241000	1.510000	0.044000	H	-2.898000	-4.629000	-0.843000
O	3.985000	-3.171000	-0.027000	H	-3.993000	-5.803000	-2.709000
H	3.807000	1.224000	-0.257000	H	-2.501000	-6.699000	-2.284000
H	4.745000	-3.702000	0.329000	H	2.881000	-2.964000	2.675000
H	-1.824000	-1.092000	0.776000	H	1.372000	-4.227000	4.112000
C	5.088000	-1.027000	0.218000	H	-1.085000	-2.662000	2.963000
H	6.048000	-1.542000	0.441000	H	-0.138000	-5.559000	2.230000
C	2.615000	-1.262000	0.482000				
H	-2.757000	4.898000	0.509000				

C	-1.195000	2.967000	0.531000
O	-0.887000	-0.764000	0.767000
H	0.991000	3.166000	0.482000
C	3.963000	-1.967000	0.690000
O	1.467000	0.822000	0.806000
H	1.345000	-2.714000	0.263000
O	5.070000	0.152000	1.009000
O	1.570000	-2.059000	0.974000
C	3.880000	0.906000	0.809000
C	-0.915000	0.567000	1.209000
H	-2.015000	3.080000	1.279000
C	2.617000	0.095000	1.211000
H	3.374000	7.376000	1.643000
H	0.117000	5.367000	1.018000
O	5.138000	2.890000	1.289000
C	0.172000	3.225000	1.232000
C	0.455000	0.954000	1.798000
H	4.086000	-2.177000	1.780000
H	-1.674000	0.683000	2.020000
H	5.253000	3.600000	1.973000
O	3.665000	6.562000	2.132000
C	3.986000	2.182000	1.652000
C	0.228000	4.632000	1.836000
H	3.097000	2.824000	1.488000
H	1.063000	6.881000	2.454000
O	0.404000	2.288000	2.283000
H	2.686000	-0.092000	2.309000
O	1.475000	4.827000	2.468000
H	0.611000	0.277000	2.668000
H	-0.604000	4.730000	2.571000
H	4.046000	1.898000	2.728000
C	1.506000	6.094000	3.109000
C	2.961000	6.542000	3.343000
H	2.967000	7.561000	3.798000
H	5.524000	5.681000	3.881000
H	3.662000	4.546000	3.896000
O	0.786000	6.075000	4.335000
C	3.637000	5.577000	4.325000
O	4.943000	6.005000	4.618000
H	1.315000	4.123000	4.846000
C	1.345000	5.155000	5.265000
C	2.807000	5.538000	5.613000
H	2.827000	6.562000	6.061000
H	-0.573000	4.845000	6.212000
C	0.456000	5.145000	6.515000
O	3.369000	4.600000	6.497000
O	0.419000	6.421000	7.093000
H	0.846000	4.407000	7.250000
H	3.266000	4.963000	7.415000
H	-0.188000	6.353000	7.875000
O	10.492000	6.880000	-0.072000
O	5.451000	5.728000	0.298000
O	9.761000	5.884000	-1.919000
O	9.305000	1.698000	2.065000
O	8.711000	3.477000	3.265000
H	-0.147000	-2.908000	0.557000
H	-0.495000	-4.677000	-1.011000
H	1.261000	-4.328000	-1.094000
H	3.606000	3.057000	1.340000
H	4.312000	1.262000	2.914000
H	1.269000	0.634000	3.310000
H	3.478000	-1.113000	1.949000
H	1.164000	0.371000	0.473000
H	2.078000	-1.604000	-0.691000
H	2.585000	-0.112000	-1.556000
H	-1.276000	5.346000	4.444000
H	1.488000	3.402000	3.988000
H	1.820000	4.786000	-2.721000
H	-6.557000	5.074000	2.585000
H	-3.251000	5.562000	3.037000
H	-1.813000	7.423000	-1.237000
H	-9.794000	1.080000	0.391000
H	-7.821000	3.691000	0.933000
H	-6.169000	4.694000	-3.601000
H	-8.876000	-4.462000	0.456000
H	-9.216000	-1.191000	-0.335000
H	-4.319000	-2.905000	-4.480000
H	-4.496000	-5.009000	3.939000
H	-6.734000	-4.619000	1.379000
H	-4.956000	-7.497000	-1.303000
H	0.196000	-1.831000	4.684000
H	-2.069000	-4.472000	4.026000
H	0.114000	-6.515000	0.142000
H	3.271000	2.098000	4.787000
H	2.247000	-1.069000	4.494000
O	9.196000	0.073000	-0.680000
C	7.956000	-0.635000	-0.642000
C	7.017000	0.124000	0.332000
O	5.729000	-0.483000	0.330000
C	5.075000	-0.453000	-0.922000
C	5.891000	-1.188000	-2.031000
C	7.338000	-0.622000	-2.071000
O	8.062000	-1.434000	-2.970000
O	5.214000	-0.923000	-3.242000
C	7.520000	0.034000	1.790000
O	6.884000	0.953000	2.633000
C	10.355000	-0.731000	-0.661000
O	10.610000	-0.930000	-2.035000
C	11.491000	0.024000	-2.629000
C	12.912000	-0.088000	-2.007000
O	13.644000	1.104000	-2.204000
C	12.866000	-0.309000	-0.464000
C	11.469000	0.037000	0.112000
O	11.374000	-0.188000	1.493000
O	13.134000	-1.662000	-0.149000

O	-0.216000	3.737000	-8.927000
N	7.192000	5.458000	-1.074000
N	2.009000	2.953000	-4.356000
N	-0.134000	2.687000	-6.401000
N	-2.114000	0.314000	-8.307000
N	-1.448000	0.311000	-6.037000
N	-1.462000	2.078000	-9.731000
N	-2.803000	0.300000	-10.618000
C	8.153000	6.069000	-0.148000
C	8.299000	5.243000	1.160000
C	8.755000	3.796000	0.905000
C	5.863000	5.324000	-0.777000
C	4.875000	4.707000	-1.725000
C	9.522000	6.263000	-0.782000
C	2.942000	3.548000	-3.454000
C	0.608000	2.766000	-4.035000
C	3.543000	4.508000	-1.300000
C	5.214000	4.317000	-3.037000
C	2.591000	3.934000	-2.149000
C	4.255000	3.752000	-3.880000
C	-0.122000	2.103000	-5.172000
C	8.920000	2.987000	2.164000
C	-0.782000	2.110000	-7.450000
C	-1.458000	0.895000	-7.264000
C	-0.795000	0.884000	-4.990000
C	-0.796000	2.691000	-8.718000
C	-2.119000	0.903000	-9.537000
H	7.790000	7.087000	0.113000
H	7.335000	5.216000	1.707000
H	9.030000	5.751000	1.827000
H	7.994000	3.291000	0.280000
H	9.727000	3.796000	0.367000
H	7.533000	5.135000	-1.985000
H	0.149000	3.758000	-3.847000
H	0.519000	2.148000	-3.115000
H	3.231000	4.789000	-0.302000
H	6.204000	4.442000	-3.445000
H	2.353000	2.653000	-5.282000
H	1.588000	3.791000	-1.778000
H	4.529000	3.472000	-4.880000
H	-0.805000	0.393000	-4.025000
H	11.374000	7.026000	-0.441000
H	-2.617000	-0.590000	-8.161000
H	9.424000	1.143000	2.849000
H	-3.307000	-0.589000	-10.491000
H	-2.807000	0.750000	-11.545000

C	11.497000	-0.310000	-4.142000
O	12.056000	0.727000	-4.903000
H	8.105000	-1.688000	-0.293000
H	6.924000	1.194000	0.019000
H	4.810000	0.590000	-1.227000
H	5.912000	-2.290000	-1.842000
H	7.328000	0.424000	-2.470000
H	8.991000	-1.231000	-2.848000
H	5.669000	-1.413000	-3.914000
H	7.421000	-0.997000	2.185000
H	8.583000	0.329000	1.857000
H	5.955000	0.738000	2.657000
H	10.176000	-1.765000	-0.276000
H	11.080000	1.053000	-2.465000
H	13.461000	-0.936000	-2.491000
H	13.718000	1.230000	-3.141000
H	13.627000	0.338000	0.043000
H	11.311000	1.141000	0.051000
H	11.592000	-1.097000	1.662000
H	14.063000	-1.788000	-0.281000
H	12.149000	-1.172000	-4.375000
H	10.477000	-0.553000	-4.501000
H	11.456000	1.461000	-4.872000
O	-4.789000	2.839000	-8.644000
O	-1.343000	2.167000	-5.028000
O	-5.811000	2.524000	-6.641000
O	-4.436000	-2.844000	-8.258000
O	-2.508000	-1.901000	-8.991000
O	-2.943000	3.028000	5.811000
N	-3.552000	1.635000	-5.489000
N	-3.319000	0.712000	0.857000
N	-2.831000	1.089000	3.979000
N	-4.279000	-0.584000	6.885000
N	-3.765000	-1.458000	4.707000
N	-3.860000	1.694000	7.443000
N	-4.793000	0.191000	8.983000
C	-3.486000	1.851000	-6.912000
C	-3.062000	0.594000	-7.682000
C	-4.009000	-0.608000	-7.574000
C	-2.458000	1.815000	-4.653000
C	-2.683000	1.525000	-3.214000
C	-4.820000	2.416000	-7.352000
C	-3.105000	0.984000	-0.510000
C	-2.272000	0.300000	1.740000
C	-1.617000	1.126000	-2.408000
C	-3.960000	1.655000	-2.668000
C	-1.828000	0.856000	-1.055000
C	-4.171000	1.384000	-1.316000
C	-2.813000	0.052000	3.121000
C	-3.540000	-1.826000	-8.340000

	C -3.325000 0.817000 5.204000
	C -3.791000 -0.431000 5.584000
	C -3.272000 -1.197000 3.478000
	C -3.360000 1.933000 6.175000
	C -4.286000 0.493000 7.744000
	H -2.740000 2.636000 -7.088000
	H -2.066000 0.284000 -7.336000
	H -2.945000 0.848000 -8.743000
	H -4.114000 -0.902000 -6.523000
	H -4.995000 -0.332000 -7.962000
	H -4.422000 1.262000 -5.120000
	H -1.500000 1.076000 1.810000
	H -1.786000 -0.616000 1.382000
	H -0.616000 1.016000 -2.816000
	H -4.810000 1.994000 -3.252000
	H -4.263000 0.828000 1.210000
	H -0.970000 0.548000 -0.465000
	H -5.170000 1.496000 -0.901000
	H -3.264000 -2.037000 2.793000
	H -5.649000 3.220000 -8.923000
	H -4.619000 -1.496000 7.161000
	H -4.145000 -3.629000 -8.768000
	H -5.120000 -0.742000 9.208000
	H -4.854000 0.895000 9.711000

Table S4. Cartesian coordinates of obtained structures of FA complexes (conformation 2)

FA/ G-β–CD	FA/ Ma-β–CD
**Atom x y z**	**Atom x y z**
2.948000 5.531000 -10.645000	C -0.944000 5.954000 1.307000
H 0.753000 5.666000 -10.783000	C -0.429000 5.614000 2.735000
H 2.177000 2.462000 -10.225000	C 0.222000 4.207000 2.765000
H 5.136000 0.900000 -10.480000	C 1.256000 4.058000 1.609000
O 3.055000 2.926000 -10.206000	C 0.655000 4.505000 0.246000
O 1.025000 4.919000 -10.189000	C 1.762000 4.654000 -0.819000
C 2.338000 5.190000 -9.774000	C -5.850000 5.121000 -0.544000
H 5.020000 4.529000 -9.537000	C -5.682000 5.548000 0.943000
O 4.783000 0.728000 -9.568000	C -4.288000 5.123000 1.469000
H 6.335000 2.887000 -9.465000	C -3.195000 5.669000 0.503000
H 1.939000 7.191000 -9.166000	C -3.481000 5.216000 -0.952000
C 2.975000 3.913000 -9.211000	C -2.532000 5.833000 -2.006000
H 6.778000 0.525000 -9.067000	C -8.062000 0.615000 -2.276000
C 2.349000 6.277000 -8.682000	C -8.772000 1.472000 -1.185000
C 4.383000 4.222000 -8.675000	C -7.729000 2.297000 -0.389000

C	5.835000	0.974000	-8.673000	C	-6.877000	3.119000	-1.398000
O	3.689000	6.553000	-8.300000	C	-6.240000	2.177000	-2.452000
C	6.034000	2.490000	-8.471000	C	-5.479000	2.914000	-3.580000
O	-1.124000	5.945000	-8.436000	C	-6.448000	-4.422000	-1.652000
H	-1.786000	5.205000	-8.438000	C	-7.823000	-3.982000	-1.067000
H	2.361000	3.542000	-8.353000	C	-7.842000	-2.457000	-0.797000
O	6.498000	6.251000	-8.241000	C	-7.398000	-1.692000	-2.079000
H	4.477000	-1.259000	-7.739000	C	-6.044000	-2.240000	-2.599000
O	4.838000	3.074000	-7.973000	C	-5.717000	-1.684000	-4.004000
H	7.367000	6.511000	-7.838000	C	-2.501000	-5.881000	1.585000
C	4.302000	5.420000	-7.692000	C	-3.890000	-5.741000	2.271000
O	1.584000	5.845000	-7.560000	C	-4.810000	-4.818000	1.434000
H	0.609000	7.748000	-7.634000	C	-4.844000	-5.291000	-0.049000
O	7.110000	2.719000	-7.571000	C	-3.427000	-5.589000	-0.618000
O	5.412000	-1.070000	-7.464000	C	-3.506000	-6.415000	-1.924000
C	5.685000	5.840000	-7.176000	C	1.791000	-2.871000	2.448000
C	5.524000	0.322000	-7.319000	C	0.906000	-3.320000	3.656000
H	6.160000	4.992000	-6.642000	C	-0.547000	-3.624000	3.209000
C	0.655000	6.794000	-7.059000	C	-0.531000	-4.548000	1.955000
C	-0.741000	6.149000	-7.101000	C	0.325000	-3.890000	0.843000
H	3.720000	5.114000	-6.798000	C	0.459000	-4.731000	-0.448000
H	-1.815000	8.017000	-6.952000	C	2.878000	2.252000	1.597000
H	4.571000	0.741000	-6.911000	C	3.231000	1.567000	2.950000
H	7.582000	0.197000	-6.724000	C	2.338000	0.324000	3.168000
H	-0.680000	5.170000	-6.567000	C	2.472000	-0.618000	1.937000
C	6.647000	0.651000	-6.323000	C	2.253000	0.141000	0.601000
C	-1.752000	7.057000	-6.386000	C	2.703000	-0.693000	-0.621000
H	5.555000	6.683000	-6.462000	O	-1.535000	5.725000	3.610000
H	3.165000	7.201000	-5.832000	O	0.825000	4.093000	4.036000
C	6.837000	2.192000	-6.275000	O	1.528000	2.660000	1.517000
O	-3.011000	6.439000	-6.360000	O	0.050000	5.802000	0.332000
O	9.192000	2.050000	-5.788000	O	1.143000	4.603000	-2.082000
H	6.809000	-3.033000	-5.705000	O	-6.750000	4.965000	1.663000
H	-3.672000	7.146000	-6.137000	O	-4.165000	5.665000	2.766000
H	5.923000	2.665000	-5.853000	O	-1.965000	5.066000	0.905000
O	2.339000	9.058000	-6.182000	O	-4.805000	5.574000	-1.362000
C	1.004000	7.140000	-5.586000	O	-2.416000	7.225000	-1.943000
H	3.236000	9.464000	-6.056000	O	-9.521000	0.588000	-0.374000
H	9.882000	2.340000	-5.138000	O	-8.457000	3.134000	0.484000
H	8.056000	3.708000	-5.337000	O	-5.804000	3.714000	-0.670000
C	2.353000	7.856000	-5.462000	O	-7.233000	1.379000	-3.107000
C	7.983000	2.598000	-5.342000	O	-6.191000	3.950000	-4.191000
O	6.032000	-2.665000	-5.207000	O	-8.034000	-4.734000	0.112000
H	8.072000	-0.939000	-5.053000	O	-9.169000	-2.142000	-0.436000
H	1.102000	6.202000	-4.993000	O	-7.187000	-0.333000	-1.697000
O	6.256000	0.171000	-5.043000	O	-6.074000	-3.665000	-2.769000
O	0.010000	7.983000	-5.013000	O	-4.402000	-1.963000	-4.400000
C	-1.253000	7.339000	-4.955000	O	-3.654000	-5.248000	3.573000
H	-3.382000	4.429000	-4.624000	O	-6.086000	-4.878000	2.036000
H	2.533000	8.070000	-4.387000	O	-5.364000	-4.182000	-0.786000

H	-1.972000	8.071000	-4.527000	O	-2.608000	-6.353000	0.269000
C	7.199000	-0.692000	-4.410000	O	-4.165000	-7.646000	-1.815000
H	7.763000	2.253000	-4.311000	O	0.997000	-2.362000	4.684000
O	-1.167000	6.126000	-4.206000	O	-1.178000	-4.253000	4.301000
C	6.547000	-2.044000	-4.059000	O	-1.867000	-4.627000	1.458000
O	-3.724000	4.912000	-3.826000	O	1.658000	-3.666000	1.303000
H	7.307000	-2.708000	-3.581000	O	0.845000	-6.060000	-0.259000
H	4.644000	-1.174000	-3.538000	O	3.072000	2.535000	3.968000
O	7.715000	-0.085000	-3.233000	O	2.807000	-0.303000	4.342000
H	-2.063000	3.977000	-3.004000	O	1.418000	-1.580000	2.012000
C	5.419000	-1.813000	-3.047000	O	2.973000	1.376000	0.512000
C	-1.838000	6.147000	-2.953000	O	3.993000	-1.257000	-0.515000
C	-2.707000	4.879000	-2.858000	H	-1.232000	7.030000	1.209000
H	-2.515000	7.021000	-2.810000	H	0.321000	6.389000	3.032000
H	0.656000	7.427000	-2.707000	H	-0.573000	3.409000	2.687000
O	4.861000	-3.047000	-2.674000	H	2.175000	4.654000	1.838000
H	5.937000	0.906000	-2.752000	H	-0.113000	3.764000	-0.094000
H	-4.948000	3.747000	-1.738000	H	2.324000	5.601000	-0.691000
C	6.686000	0.216000	-2.296000	H	2.489000	3.820000	-0.723000
H	3.899000	-2.887000	-2.489000	H	-6.751000	5.579000	-1.017000
H	-0.119000	5.263000	-1.903000	H	-5.792000	6.660000	1.006000
C	5.981000	-1.083000	-1.815000	H	-4.218000	4.007000	1.538000
C	-0.814000	6.127000	-1.785000	H	-3.144000	6.786000	0.576000
C	0.068000	7.379000	-1.770000	H	-3.370000	4.097000	-1.024000
H	-0.577000	8.282000	-1.673000	H	-1.540000	5.342000	-1.928000
O	-4.063000	3.595000	-1.315000	H	-2.922000	5.658000	-3.026000
C	-3.333000	4.785000	-1.456000	H	-8.789000	0.161000	-2.991000
H	-2.563000	1.310000	-1.291000	H	-9.493000	2.159000	-1.694000
H	-4.000000	5.664000	-1.284000	H	-7.074000	1.619000	0.213000
H	6.736000	-1.756000	-1.347000	H	-7.518000	3.899000	-1.884000
O	7.950000	2.133000	-1.606000	H	-5.507000	1.496000	-1.947000
O	4.888000	-0.766000	-0.969000	H	-4.511000	3.278000	-3.176000
C	7.319000	0.978000	-1.127000	H	-5.264000	2.225000	-4.417000
H	8.067000	0.324000	-0.625000	H	-6.481000	-5.463000	-2.051000
H	-0.536000	1.263000	-0.615000	H	-8.618000	-4.250000	-1.808000
O	-2.582000	1.159000	-0.310000	H	-7.159000	-2.211000	0.055000
O	0.955000	7.315000	-0.687000	H	-8.186000	-1.770000	-2.872000
H	8.338000	2.585000	-0.812000	H	-5.223000	-1.972000	-1.887000
O	-1.494000	6.055000	-0.536000	H	-6.440000	-2.046000	-4.761000
H	1.501000	8.143000	-0.728000	H	-5.746000	-0.579000	-3.997000
O	-1.343000	3.722000	-0.531000	H	-1.862000	-6.656000	2.074000
H	2.463000	-1.179000	-0.400000	H	-4.343000	-6.759000	2.362000
C	-2.217000	4.841000	-0.397000	H	-4.450000	-3.760000	1.496000
H	6.529000	1.274000	-0.410000	H	-5.488000	-6.205000	-0.146000
C	-1.297000	1.446000	0.180000	H	-2.898000	-4.629000	-0.843000
O	4.011000	-3.238000	0.190000	H	-3.993000	-5.803000	-2.709000
H	3.755000	1.118000	-0.112000	H	-2.501000	-6.699000	-2.284000
H	4.789000	-3.746000	0.537000	H	2.881000	-2.964000	2.675000
H	-1.906000	-1.124000	0.914000	H	1.372000	-4.227000	4.112000
C	5.072000	-1.071000	0.414000	H	-1.085000	-2.662000	2.963000

H	6.046000	-1.562000	0.632000	H	-0.138000	-5.559000	2.230000
C	2.607000	-1.352000	0.691000	H	-0.147000	-2.908000	0.557000
H	-2.726000	4.879000	0.592000	H	-0.495000	-4.677000	-1.011000
C	-1.212000	2.914000	0.631000	H	1.261000	-4.328000	-1.094000
O	-0.961000	-0.821000	0.940000	H	3.606000	3.057000	1.340000
H	0.968000	3.038000	0.529000	H	4.312000	1.262000	2.914000
C	3.969000	-2.029000	0.901000	H	1.269000	0.634000	3.310000
O	1.415000	0.708000	0.951000	H	3.478000	-1.113000	1.949000
H	1.335000	-2.803000	0.477000	H	1.164000	0.371000	0.473000
O	5.026000	0.113000	1.195000	H	2.078000	-1.604000	-0.691000
O	1.573000	-2.158000	1.193000	H	2.585000	-0.112000	-1.556000
C	3.828000	0.843000	0.963000	H	-1.276000	5.346000	4.444000
C	-0.971000	0.518000	1.359000	H	1.488000	3.402000	3.988000
H	-2.014000	3.066000	1.390000	H	1.820000	4.786000	-2.721000
C	2.578000	0.022000	1.387000	H	-6.557000	5.074000	2.585000
H	3.715000	6.987000	1.417000	H	-3.251000	5.562000	3.037000
H	0.195000	5.282000	1.000000	H	-1.813000	7.423000	-1.237000
O	5.046000	2.875000	1.308000	H	-9.794000	1.080000	0.391000
C	0.172000	3.151000	1.297000	H	-7.821000	3.691000	0.933000
C	0.408000	0.894000	1.936000	H	-6.169000	4.694000	-3.601000
H	4.100000	-2.229000	1.992000	H	-8.876000	-4.462000	0.456000
H	-1.724000	0.658000	2.171000	H	-9.216000	-1.191000	-0.335000
H	5.087000	3.692000	1.869000	H	-4.319000	-2.905000	-4.480000
O	3.929000	6.203000	1.987000	H	-4.496000	-5.009000	3.939000
C	3.919000	2.161000	1.735000	H	-6.734000	-4.619000	1.379000
C	0.301000	4.575000	1.844000	H	-4.956000	-7.497000	-1.303000
H	3.014000	2.767000	1.541000	H	0.196000	-1.831000	4.684000
H	1.355000	6.801000	2.238000	H	-2.069000	-4.472000	4.026000
O	0.390000	2.244000	2.377000	H	0.114000	-6.515000	0.142000
H	2.643000	-0.131000	2.490000	H	3.271000	2.098000	4.787000
O	1.576000	4.731000	2.427000	H	2.247000	-1.069000	4.494000
H	0.553000	0.237000	2.824000	O	9.196000	0.073000	-0.680000
H	-0.501000	4.737000	2.600000	C	7.956000	-0.635000	-0.642000
H	4.001000	1.944000	2.824000	C	7.017000	0.124000	0.332000
C	1.718000	6.036000	2.963000	O	5.729000	-0.483000	0.330000
C	3.209000	6.362000	3.183000	C	5.075000	-0.453000	-0.922000
H	3.307000	7.413000	3.547000	C	5.891000	-1.188000	-2.031000
H	5.675000	5.339000	3.857000	C	7.338000	-0.622000	-2.071000
H	3.720000	4.370000	3.923000	O	8.062000	-1.434000	-2.970000
O	0.989000	6.182000	4.176000	O	5.214000	-0.923000	-3.242000
C	3.780000	5.432000	4.263000	C	7.520000	0.034000	1.790000
O	5.112000	5.769000	4.552000	O	6.884000	0.953000	2.633000
H	1.338000	4.230000	4.832000	C	10.355000	-0.731000	-0.661000
C	1.447000	5.284000	5.179000	O	10.610000	-0.930000	-2.035000
C	2.929000	5.568000	5.532000	C	11.491000	0.024000	-2.629000
H	3.030000	6.619000	5.900000	C	12.912000	-0.088000	-2.007000
H	-0.508000	5.193000	6.096000	O	13.644000	1.104000	-2.204000
C	0.535000	5.428000	6.405000	C	12.866000	-0.309000	-0.464000
O	3.394000	4.659000	6.498000	C	11.469000	0.037000	0.112000
O	0.593000	6.737000	6.902000	O	11.374000	-0.188000	1.493000

H	0.849000	4.708000	7.193000	O	13.134000	-1.662000	-0.149000
H	3.295000	5.095000	7.384000	C	11.497000	-0.310000	-4.142000
H	-0.024000	6.764000	7.679000	O	12.056000	0.727000	-4.903000
O	-0.444000	-4.315000	-5.407000	H	8.105000	-1.688000	-0.293000
O	0.759000	-0.483000	-2.137000	H	6.924000	1.194000	0.019000
O	0.678000	-2.703000	-6.448000	H	4.810000	0.590000	-1.227000
O	-4.309000	-0.079000	-4.988000	H	5.912000	-2.290000	-1.842000
O	-3.940000	-1.315000	-3.174000	H	7.328000	0.424000	-2.470000
O	8.885000	6.301000	-4.802000	H	8.991000	-1.231000	-2.848000
N	0.894000	-1.014000	-4.304000	H	5.669000	-1.413000	-3.914000
N	4.118000	4.748000	-3.675000	H	7.421000	-0.997000	2.185000
N	6.454000	6.572000	-3.565000	H	8.583000	0.329000	1.857000
N	7.541000	9.981000	-4.163000	H	5.955000	0.738000	2.657000
N	5.579000	9.135000	-3.153000	H	10.176000	-1.765000	-0.276000
N	9.188000	8.503000	-4.983000	H	11.080000	1.053000	-2.465000
N	9.533000	10.869000	-5.187000	H	13.461000	-0.936000	-2.491000
C	0.149000	-2.267000	-4.149000	H	13.718000	1.230000	-3.141000
C	-1.311000	-2.005000	-3.692000	H	13.627000	0.338000	0.043000
C	-2.099000	-1.118000	-4.675000	H	11.311000	1.141000	0.051000
C	1.153000	-0.177000	-3.253000	H	11.592000	-1.097000	1.662000
C	1.920000	1.104000	-3.405000	H	14.063000	-1.788000	-0.281000
C	0.147000	-3.101000	-5.420000	H	12.149000	-1.172000	-4.375000
C	3.373000	3.537000	-3.565000	H	10.477000	-0.553000	-4.501000
C	4.424000	5.595000	-2.537000	H	11.456000	1.461000	-4.872000
C	2.129000	1.907000	-2.269000	O	-1.085000	2.869000	8.702000
C	2.449000	1.550000	-4.634000	O	-4.531000	2.197000	5.086000
C	2.844000	3.101000	-2.341000	O	-0.064000	2.554000	6.700000
C	3.162000	2.750000	-4.707000	O	-1.438000	-2.815000	8.316000
C	5.251000	6.776000	-2.961000	O	-3.367000	-1.872000	9.050000
C	-3.511000	-0.850000	-4.221000	O	-2.931000	3.057000	-5.753000
C	7.225000	7.615000	-3.971000	N	-2.322000	1.664000	5.547000
C	6.777000	8.930000	-3.761000	N	-2.556000	0.741000	-0.799000
C	4.807000	8.091000	-2.749000	N	-3.043000	1.118000	-3.921000
C	8.458000	7.423000	-4.598000	N	-1.595000	-0.554000	-6.827000
C	8.743000	9.772000	-4.772000	N	-2.109000	-1.429000	-4.649000
H	0.659000	-2.884000	-3.377000	N	-2.014000	1.724000	-7.385000
H	-1.317000	-1.517000	-2.697000	N	-1.082000	0.220000	-8.925000
H	-1.833000	-2.980000	-3.571000	C	-2.388000	1.880000	6.971000
H	-1.584000	-0.140000	-4.782000	C	-2.812000	0.623000	7.740000
H	-2.141000	-1.610000	-5.670000	C	-1.865000	-0.578000	7.632000
H	1.250000	-0.769000	-5.234000	C	-3.416000	1.844000	4.712000
H	5.005000	5.012000	-1.790000	C	-3.191000	1.555000	3.272000
H	3.476000	5.937000	-2.067000	C	-1.055000	2.446000	7.411000
H	1.739000	1.612000	-1.312000	C	-2.769000	1.013000	0.569000
H	2.332000	0.997000	-5.551000	C	-3.603000	0.329000	-1.682000
H	4.470000	5.018000	-4.605000	C	-4.258000	1.155000	2.466000
H	2.977000	3.673000	-1.433000	C	-1.915000	1.684000	2.726000
H	3.555000	3.062000	-5.662000	C	-4.046000	0.885000	1.114000
H	3.857000	8.286000	-2.268000	C	-1.703000	1.413000	1.374000
H	-0.462000	-4.878000	-6.194000	C	-3.061000	0.081000	-3.062000

H	7.201000	10.956000	-4.002000	C	-2.334000	-1.797000	8.399000
H	-5.221000	0.118000	-4.730000	C	-2.549000	0.846000	-5.146000
H	9.212000	11.835000	-5.034000	C	-2.083000	-0.402000	-5.525000
H	10.442000	10.710000	-5.646000	C	-2.602000	-1.168000	-3.420000
				C	-2.514000	1.962000	-6.117000
				C	-1.588000	0.522000	-7.685000
				H	-3.135000	2.665000	7.147000
				H	-3.809000	0.313000	7.395000
				H	-2.929000	0.877000	8.802000
				H	-1.761000	-0.872000	6.582000
				H	-0.879000	-0.303000	8.021000
				H	-1.453000	1.291000	5.178000
				H	-4.375000	1.105000	-1.752000
				H	-4.088000	-0.586000	-1.324000
				H	-5.258000	1.045000	2.874000
				H	-1.064000	2.023000	3.311000
				H	-1.611000	0.857000	-1.151000
				H	-4.904000	0.577000	0.523000
				H	-0.704000	1.525000	0.960000
				H	-2.610000	-2.008000	-2.734000
				H	-0.225000	3.249000	8.981000
				H	-1.255000	-1.467000	-7.103000
				H	-1.729000	-3.600000	8.826000
				H	-0.754000	-0.713000	-9.150000
				H	-1.021000	0.924000	-9.653000

Table S5. Cartesian coordinates of obtained structures of MTX complexes (conformation 1)

MTX/ G-β–CD				MTX/ Ma-β–CD			
**Atom**	**x**	**y**	**z**	**Atom**	**x**	**y**	**z**
H	2.810000	5.512000	-10.627000	C	-0.944000	5.954000	1.307000
H	0.603000	5.609000	-10.706000	C	-0.429000	5.614000	2.735000
H	2.045000	2.483000	-10.128000	C	0.222000	4.207000	2.765000
H	5.045000	1.000000	-10.573000	C	1.256000	4.058000	1.609000
O	2.919000	2.908000	-10.162000	C	0.655000	4.505000	0.246000
O	0.910000	4.884000	-10.139000	C	1.762000	4.654000	-0.819000
C	2.246000	5.193000	-9.746000	C	-5.850000	5.121000	-0.544000
H	4.943000	4.514000	-9.568000	C	-5.682000	5.548000	0.943000
O	4.801000	0.663000	-9.696000	C	-4.288000	5.123000	1.469000
H	6.299000	2.978000	-9.550000	C	-3.195000	5.669000	0.503000
H	1.865000	7.262000	-9.136000	C	-3.481000	5.216000	-0.952000
C	2.897000	3.929000	-9.166000	C	-2.532000	5.833000	-2.006000
H	6.794000	0.545000	-9.187000	C	-8.062000	0.615000	-2.276000
C	2.267000	6.334000	-8.701000	C	-8.772000	1.472000	-1.185000
C	4.327000	4.247000	-8.705000	C	-7.729000	2.297000	-0.389000

C	5.864000	0.961000	-8.791000
O	3.633000	6.561000	-8.284000
C	6.037000	2.488000	-8.599000
O	-1.144000	6.565000	-8.501000
H	-1.807000	5.851000	-8.588000
H	2.314000	3.579000	-8.309000
O	6.426000	6.361000	-8.393000
H	4.448000	-1.204000	-7.858000
O	4.819000	3.053000	-8.058000
H	7.095000	6.979000	-8.062000
C	4.302000	5.413000	-7.687000
O	1.490000	5.957000	-7.543000
H	0.871000	7.969000	-7.449000
O	7.110000	2.706000	-7.653000
O	5.379000	-1.091000	-7.599000
C	5.710000	5.862000	-7.262000
C	5.554000	0.312000	-7.434000
H	6.256000	5.029000	-6.814000
C	0.698000	7.011000	-6.951000
C	-0.766000	6.583000	-7.122000
H	3.748000	5.101000	-6.797000
H	-1.666000	8.506000	-6.740000
H	4.627000	0.732000	-7.038000
H	7.613000	0.127000	-6.809000
H	-0.855000	5.573000	-6.742000
C	6.696000	0.604000	-6.451000
C	-1.703000	7.497000	-6.319000
H	5.618000	6.658000	-6.519000
H	3.214000	7.102000	-5.710000
C	6.893000	2.136000	-6.331000
O	-3.032000	6.995000	-6.420000
O	9.285000	1.962000	-5.986000
H	6.797000	-3.008000	-5.855000
H	-3.606000	7.654000	-6.010000
H	5.993000	2.582000	-5.904000
O	2.515000	9.051000	-5.747000
C	1.059000	7.118000	-5.447000
H	3.428000	9.368000	-5.653000
H	10.006000	2.259000	-5.414000
H	8.179000	3.604000	-5.407000
C	2.458000	7.723000	-5.225000
C	8.088000	2.516000	-5.445000
O	6.053000	-2.792000	-5.283000
H	8.131000	-0.953000	-5.144000
H	1.025000	6.124000	-4.992000
O	6.295000	0.066000	-5.171000
O	0.103000	7.977000	-4.767000
C	-1.277000	7.556000	-4.831000
H	-3.368000	4.661000	-4.880000
H	2.668000	7.755000	-4.155000

C	-6.877000	3.119000	-1.398000
C	-6.240000	2.177000	-2.452000
C	-5.479000	2.914000	-3.580000
C	-6.448000	-4.422000	-1.652000
C	-7.823000	-3.982000	-1.067000
C	-7.842000	-2.457000	-0.797000
C	-7.398000	-1.692000	-2.079000
C	-6.044000	-2.240000	-2.599000
C	-5.717000	-1.684000	-4.004000
C	-2.501000	-5.881000	1.585000
C	-3.890000	-5.741000	2.271000
C	-4.810000	-4.818000	1.434000
C	-4.844000	-5.291000	-0.049000
C	-3.427000	-5.589000	-0.618000
C	-3.506000	-6.415000	-1.924000
C	1.791000	-2.871000	2.448000
C	0.906000	-3.320000	3.656000
C	-0.547000	-3.624000	3.209000
C	-0.531000	-4.548000	1.955000
C	0.325000	-3.890000	0.843000
C	0.459000	-4.731000	-0.448000
C	2.878000	2.252000	1.597000
C	3.231000	1.567000	2.950000
C	2.338000	0.324000	3.168000
C	2.472000	-0.618000	1.937000
C	2.253000	0.141000	0.601000
C	2.703000	-0.693000	-0.621000
O	-1.535000	5.725000	3.610000
O	0.825000	4.093000	4.036000
O	1.528000	2.660000	1.517000
O	0.050000	5.802000	0.332000
O	1.143000	4.603000	-2.082000
O	-6.750000	4.965000	1.663000
O	-4.165000	5.665000	2.766000
O	-1.965000	5.066000	0.905000
O	-4.805000	5.574000	-1.362000
O	-2.416000	7.225000	-1.943000
O	-9.521000	0.588000	-0.374000
O	-8.457000	3.134000	0.484000
O	-5.804000	3.714000	-0.670000
O	-7.233000	1.379000	-3.107000
O	-6.191000	3.950000	-4.191000
O	-8.034000	-4.734000	0.112000
O	-9.169000	-2.142000	-0.436000
O	-7.187000	-0.333000	-1.697000
O	-6.074000	-3.665000	-2.769000
O	-4.402000	-1.963000	-4.400000
O	-3.654000	-5.248000	3.573000
O	-6.086000	-4.878000	2.036000
O	-5.364000	-4.182000	-0.786000

H	-1.885000	8.294000	-4.285000	O	-2.608000	-6.353000	0.269000
C	7.250000	-0.793000	-4.503000	O	-4.165000	-7.646000	-1.815000
H	7.934000	2.146000	-4.428000	O	0.997000	-2.362000	4.684000
O	-1.424000	6.251000	-4.215000	O	-1.178000	-4.253000	4.301000
C	6.572000	-2.135000	-4.133000	O	-1.867000	-4.627000	1.458000
O	-3.872000	4.812000	-4.047000	O	1.658000	-3.666000	1.303000
H	7.307000	-2.787000	-3.653000	O	0.845000	-6.060000	-0.259000
H	4.671000	-1.238000	-3.658000	O	3.072000	2.535000	3.968000
O	7.689000	-0.155000	-3.278000	O	2.807000	-0.303000	4.342000
H	-2.243000	4.024000	-3.068000	O	1.418000	-1.580000	2.012000
C	5.411000	-1.877000	-3.167000	O	2.973000	1.376000	0.512000
C	-2.152000	6.186000	-2.963000	O	3.993000	-1.257000	-0.515000
C	-2.940000	4.854000	-2.955000	H	-1.232000	7.030000	1.209000
H	-2.858000	7.018000	-2.896000	H	0.321000	6.389000	3.032000
H	0.349000	7.591000	-2.382000	H	-0.573000	3.409000	2.687000
O	4.789000	-3.109000	-2.811000	H	2.175000	4.654000	1.838000
H	5.902000	0.816000	-2.802000	H	-0.113000	3.764000	-0.094000
H	-5.098000	3.520000	-2.119000	H	2.324000	5.601000	-0.691000
C	6.632000	0.154000	-2.326000	H	2.489000	3.820000	-0.723000
H	3.863000	-2.887000	-2.617000	H	-6.751000	5.579000	-1.017000
H	-0.454000	5.390000	-1.866000	H	-5.792000	6.660000	1.006000
C	5.945000	-1.170000	-1.912000	H	-4.218000	4.007000	1.538000
C	-1.174000	6.204000	-1.754000	H	-3.144000	6.786000	0.576000
C	-0.395000	7.516000	-1.588000	H	-3.370000	4.097000	-1.024000
H	-1.073000	8.372000	-1.648000	H	-1.540000	5.342000	-1.928000
O	-4.339000	3.446000	-1.526000	H	-2.922000	5.658000	-3.026000
C	-3.653000	4.690000	-1.593000	H	-8.789000	0.161000	-2.991000
H	-2.707000	1.287000	-1.498000	H	-9.493000	2.159000	-1.694000
H	-4.372000	5.503000	-1.455000	H	-7.074000	1.619000	0.213000
H	6.657000	-1.827000	-1.404000	H	-7.518000	3.899000	-1.884000
O	7.961000	2.042000	-1.592000	H	-5.507000	1.496000	-1.947000
O	4.804000	-0.909000	-1.072000	H	-4.511000	3.278000	-3.176000
C	7.259000	0.889000	-1.130000	H	-5.264000	2.225000	-4.417000
H	7.950000	0.225000	-0.605000	H	-6.481000	-5.463000	-2.051000
H	-0.683000	1.197000	-0.708000	H	-8.618000	-4.250000	-1.808000
O	-2.723000	0.979000	-0.568000	H	-7.159000	-2.211000	0.055000
O	0.284000	7.526000	-0.324000	H	-8.186000	-1.770000	-2.872000
H	8.346000	2.465000	-0.808000	H	-5.223000	-1.972000	-1.887000
O	-1.929000	5.996000	-0.531000	H	-6.440000	-2.046000	-4.761000
H	-0.253000	8.047000	0.305000	H	-5.746000	-0.579000	-3.997000
O	-1.675000	3.640000	-0.617000	H	-1.862000	-6.656000	2.074000
H	2.341000	-1.276000	-0.521000	H	-4.343000	-6.759000	2.362000
C	-2.618000	4.733000	-0.452000	H	-4.450000	-3.760000	1.496000
H	6.475000	1.210000	-0.441000	H	-5.488000	-6.205000	-0.146000
C	-1.472000	1.327000	0.038000	H	-2.898000	-4.629000	-0.843000
O	3.877000	-3.334000	0.121000	H	-3.993000	-5.803000	-2.709000
H	3.550000	1.047000	-0.243000	H	-2.501000	-6.699000	-2.284000
H	4.707000	-3.757000	0.376000	H	2.881000	-2.964000	2.675000
H	-2.089000	-1.170000	0.560000	H	1.372000	-4.227000	4.112000
C	4.963000	-1.166000	0.343000	H	-1.085000	-2.662000	2.963000

H	5.951000	-1.605000	0.547000	H	-0.138000	-5.559000	2.230000
C	2.468000	-1.424000	0.555000	H	-0.147000	-2.908000	0.557000
H	-3.114000	4.665000	0.528000	H	-0.495000	-4.677000	-1.011000
C	-1.467000	2.791000	0.534000	H	1.261000	-4.328000	-1.094000
O	-1.183000	-0.957000	0.824000	H	3.606000	3.057000	1.340000
H	0.692000	2.995000	0.428000	H	4.312000	1.262000	2.914000
C	3.824000	-2.094000	0.818000	H	1.269000	0.634000	3.310000
O	1.211000	0.603000	0.831000	H	3.478000	-1.113000	1.949000
H	0.892000	-2.508000	0.241000	H	1.164000	0.371000	0.473000
O	4.851000	0.073000	1.071000	H	2.078000	-1.604000	-0.691000
O	1.422000	-2.271000	1.026000	H	2.585000	-0.112000	-1.556000
C	3.624000	0.814000	0.824000	H	-1.276000	5.346000	4.444000
C	-1.182000	0.405000	1.239000	H	1.488000	3.402000	3.988000
H	-2.271000	2.926000	1.263000	H	1.820000	4.786000	-2.721000
C	2.420000	-0.053000	1.257000	H	-6.557000	5.074000	2.585000
H	3.397000	6.204000	1.195000	H	-3.251000	5.562000	3.037000
H	-0.154000	5.268000	1.018000	H	-1.813000	7.423000	-1.237000
O	4.888000	2.820000	1.232000	H	-9.794000	1.080000	0.391000
C	-0.091000	3.103000	1.184000	H	-7.821000	3.691000	0.933000
C	0.191000	0.765000	1.842000	H	-6.169000	4.694000	-3.601000
H	3.936000	-2.281000	1.890000	H	-8.876000	-4.462000	0.456000
H	-1.954000	0.542000	2.001000	H	-9.216000	-1.191000	-0.335000
H	5.090000	3.446000	1.943000	H	-4.319000	-2.905000	-4.480000
O	3.885000	5.772000	1.920000	H	-4.496000	-5.009000	3.939000
C	3.700000	2.129000	1.611000	H	-6.734000	-4.619000	1.379000
C	0.019000	4.513000	1.786000	H	-4.956000	-7.497000	-1.303000
H	2.829000	2.749000	1.390000	H	0.196000	-1.831000	4.684000
H	1.321000	6.737000	2.199000	H	-2.069000	-4.472000	4.026000
O	0.156000	2.150000	2.256000	H	0.114000	-6.515000	0.142000
H	2.436000	-0.182000	2.343000	H	3.271000	2.098000	4.787000
O	1.369000	4.666000	2.283000	H	2.247000	-1.069000	4.494000
H	0.410000	0.128000	2.713000	O	9.196000	0.073000	-0.680000
H	-0.695000	4.640000	2.603000	C	7.956000	-0.635000	-0.642000
H	3.725000	1.917000	2.683000	C	7.017000	0.124000	0.332000
C	1.660000	5.946000	2.886000	O	5.729000	-0.483000	0.330000
C	3.186000	6.065000	3.133000	C	5.075000	-0.453000	-0.922000
H	3.406000	7.092000	3.432000	C	5.891000	-1.188000	-2.031000
H	5.568000	4.985000	3.820000	C	7.338000	-0.622000	-2.071000
H	3.516000	4.081000	3.955000	O	8.062000	-1.434000	-2.970000
O	0.941000	6.095000	4.136000	O	5.214000	-0.923000	-3.242000
C	3.657000	5.121000	4.262000	C	7.520000	0.034000	1.790000
O	5.042000	5.352000	4.550000	O	6.884000	0.953000	2.633000
H	1.161000	4.156000	4.862000	C	10.355000	-0.731000	-0.661000
C	1.323000	5.184000	5.202000	O	10.610000	-0.930000	-2.035000
C	2.821000	5.398000	5.525000	C	11.491000	0.024000	-2.629000
H	2.981000	6.436000	5.830000	C	12.912000	-0.088000	-2.007000
H	-0.627000	5.321000	6.135000	O	13.644000	1.104000	-2.204000
C	0.419000	5.461000	6.419000	C	12.866000	-0.309000	-0.464000
O	3.232000	4.538000	6.593000	C	11.469000	0.037000	0.112000
O	0.612000	6.799000	6.882000	O	11.374000	-0.188000	1.493000

H	0.659000	4.759000	7.222000	O	13.134000	-1.662000	-0.149000
H	3.249000	5.081000	7.404000	C	11.497000	-0.310000	-4.142000
H	0.086000	6.913000	7.689000	O	12.056000	0.727000	-4.903000
O	5.313000	9.010000	1.452000	H	8.105000	-1.688000	-0.293000
O	5.077000	7.484000	-2.528000	H	6.924000	1.194000	0.019000
O	3.121000	8.955000	1.784000	H	4.810000	0.590000	-1.227000
O	-0.396000	11.503000	-0.923000	H	5.912000	-2.290000	-1.842000
O	1.427000	12.584000	-1.575000	H	7.328000	0.424000	-2.470000
N	2.170000	1.901000	-3.817000	H	8.991000	-1.231000	-2.848000
N	3.454000	7.731000	-1.017000	H	5.669000	-1.413000	-3.914000
N	-0.611000	0.837000	-5.536000	H	7.421000	-0.997000	2.185000
N	-1.400000	3.509000	-5.712000	H	8.583000	0.329000	1.857000
N	-2.688000	3.014000	-7.601000	H	5.955000	0.738000	2.657000
N	-2.801000	0.773000	-8.465000	H	10.176000	-1.765000	-0.276000
N	-1.669000	-0.960000	-7.467000	H	11.080000	1.053000	-2.465000
N	-3.932000	2.518000	-9.477000	H	13.461000	-0.936000	-2.491000
C	3.939000	9.039000	-0.493000	H	13.718000	1.230000	-3.141000
C	3.179000	10.303000	-1.020000	H	13.627000	0.338000	0.043000
C	2.617000	3.127000	-3.321000	H	11.311000	1.141000	0.051000
C	1.636000	10.275000	-0.848000	H	11.592000	-1.097000	1.662000
C	0.823000	1.340000	-3.579000	H	14.063000	-1.788000	-0.281000
C	3.560000	5.660000	-2.391000	H	12.149000	-1.172000	-4.375000
C	4.093000	7.020000	-1.983000	H	10.477000	-0.553000	-4.501000
C	1.945000	3.836000	-2.310000	H	11.456000	1.461000	-4.872000
C	3.792000	3.704000	-3.823000	O	-2.017000	1.482000	-6.569000
C	4.068000	9.030000	1.029000	O	-4.350000	0.163000	-3.187000
C	2.411000	5.074000	-1.848000	O	-0.788000	1.532000	-4.768000
C	4.247000	4.941000	-3.374000	O	-1.460000	6.971000	-4.358000
C	-0.150000	1.768000	-4.667000	O	-3.091000	6.562000	-5.741000
C	2.905000	1.190000	-4.880000	N	-2.028000	-0.386000	2.840000
C	0.901000	11.583000	-1.135000	N	-3.103000	2.084000	-3.104000
C	-0.538000	3.095000	-4.769000	N	-1.673000	1.764000	5.531000
C	-1.460000	1.259000	-6.515000	N	-2.647000	3.895000	3.969000
C	-1.859000	2.612000	-6.610000	N	-3.209000	5.103000	5.936000
C	-1.975000	0.372000	-7.475000	N	-2.781000	4.135000	8.136000
C	-3.112000	2.084000	-8.478000	N	-1.670000	2.113000	8.454000
H	4.968000	9.164000	-0.843000	N	-3.645000	6.337000	7.904000
H	3.402000	10.423000	-2.083000	C	-3.137000	2.297000	-4.572000
H	3.583000	11.183000	-0.514000	C	-3.320000	3.786000	-4.900000
H	1.377000	9.955000	0.163000	C	-2.458000	-0.003000	1.521000
H	1.251000	9.538000	-1.547000	C	-2.183000	4.677000	-4.422000
H	2.623000	7.346000	-0.591000	C	-1.054000	0.497000	3.535000
H	0.892000	0.248000	-3.559000	C	-3.190000	0.576000	-1.130000
H	0.441000	1.633000	-2.602000	C	-3.590000	0.862000	-2.533000
H	1.040000	3.441000	-1.861000	C	-1.615000	0.761000	0.694000
H	4.380000	3.206000	-4.585000	C	-3.669000	-0.479000	0.991000
H	1.862000	5.578000	-1.060000	C	-1.884000	1.752000	-5.254000
H	5.161000	5.341000	-3.805000	C	-1.980000	1.045000	-0.611000
H	2.392000	0.268000	-5.171000	C	-4.026000	-0.194000	-0.319000
H	3.906000	0.928000	-4.531000	C	-1.650000	1.709000	4.201000

H	2.984000	1.826000	-5.767000	C	-3.042000	-0.974000	3.743000
H	-0.166000	3.857000	-4.090000	C	-2.327000	6.093000	-4.917000
H	5.354000	8.960000	2.434000	C	-2.145000	2.806000	3.415000
H	-0.835000	12.365000	-1.127000	C	-2.181000	2.894000	6.143000
H	-2.218000	-1.581000	-8.050000	C	-2.681000	3.971000	5.365000
H	-1.222000	-1.344000	-6.642000	C	-2.236000	3.031000	7.577000
H	-4.108000	1.898000	-10.259000	C	-3.228000	5.152000	7.297000
H	-4.012000	3.516000	-9.633000	H	-4.028000	1.749000	-4.979000
				H	-4.276000	4.138000	-4.462000
				H	-3.441000	3.889000	-5.998000
				H	-1.203000	4.287000	-4.768000
				H	-2.128000	4.691000	-3.314000
				H	-2.250000	2.399000	-2.678000
				H	-0.521000	-0.144000	4.267000
				H	-0.293000	0.834000	2.794000
				H	-0.650000	1.126000	1.079000
				H	-4.337000	-1.096000	1.610000
				H	-1.309000	1.646000	-1.244000
				H	-4.971000	-0.580000	-0.722000
				H	-2.524000	-1.552000	4.519000
				H	-3.700000	-1.657000	3.182000
				H	-3.674000	-0.220000	4.233000
				H	-2.123000	2.767000	2.316000
				H	-1.200000	1.139000	-6.918000
				H	-1.602000	7.841000	-4.717000
				H	-1.969000	2.159000	9.400000
				H	-1.563000	1.192000	8.100000
				H	-3.943000	6.257000	8.848000
				H	-4.215000	6.930000	7.346000

Table S6. Cartesian coordinates of obtained structures of MTX complexes (conformation 2)

MTX/ G-β–CD				MTX/ Ma-β–CD			
**Atom**	**x**	**y**	**z**	**Atom**	**x**	**y**	**z**
H	2.720000	5.437000	-10.788000	C	-0.944000	5.954000	1.307000
H	0.393000	5.497000	-10.475000	C	-0.429000	5.614000	2.735000
H	2.081000	2.644000	-10.465000	C	0.222000	4.207000	2.765000
H	4.849000	1.328000	-10.411000	C	1.256000	4.058000	1.609000
O	2.972000	2.833000	-10.194000	C	0.655000	4.505000	0.246000
O	0.865000	4.756000	-10.114000	C	1.762000	4.654000	-0.819000
C	2.227000	5.135000	-9.852000	C	-5.850000	5.121000	-0.544000
H	5.018000	4.530000	-9.659000	C	-5.682000	5.548000	0.943000
O	4.630000	0.916000	-9.583000	C	-4.288000	5.123000	1.469000
H	6.335000	2.912000	-9.521000	C	-3.195000	5.669000	0.503000
H	1.828000	7.188000	-9.364000	C	-3.481000	5.216000	-0.952000
C	2.969000	3.929000	-9.272000	C	-2.532000	5.833000	-2.006000
H	6.611000	0.533000	-9.124000	C	-8.062000	0.615000	-2.276000
C	2.259000	6.299000	-8.881000	C	-8.772000	1.472000	-1.185000

C	4.382000	4.283000	-8.797000	C	-7.729000	2.297000	-0.389000
C	5.730000	1.030000	-8.692000	C	-6.877000	3.119000	-1.398000
O	3.629000	6.518000	-8.560000	C	-6.240000	2.177000	-2.452000
C	6.040000	2.511000	-8.540000	C	-5.479000	2.914000	-3.580000
O	-1.079000	5.979000	-8.419000	C	-6.448000	-4.422000	-1.652000
H	-1.947000	5.592000	-8.406000	C	-7.823000	-3.982000	-1.067000
H	2.408000	3.615000	-8.380000	C	-7.842000	-2.457000	-0.797000
O	6.034000	7.007000	-8.289000	C	-7.398000	-1.692000	-2.079000
H	4.560000	-1.214000	-8.115000	C	-6.044000	-2.240000	-2.599000
O	4.900000	3.189000	-8.059000	C	-5.717000	-1.684000	-4.004000
H	6.868000	7.362000	-8.004000	C	-2.501000	-5.881000	1.585000
C	4.276000	5.469000	-7.866000	C	-3.890000	-5.741000	2.271000
O	1.488000	5.944000	-7.704000	C	-4.810000	-4.818000	1.434000
H	0.669000	7.881000	-7.688000	C	-4.844000	-5.291000	-0.049000
O	7.089000	2.706000	-7.599000	C	-3.427000	-5.589000	-0.618000
O	5.285000	-1.039000	-7.526000	C	-3.506000	-6.415000	-1.924000
C	5.603000	5.997000	-7.366000	C	1.791000	-2.871000	2.448000
C	5.425000	0.382000	-7.346000	C	0.906000	-3.320000	3.656000
H	6.342000	5.183000	-7.327000	C	-0.547000	-3.624000	3.209000
C	0.660000	6.941000	-7.117000	C	-0.531000	-4.548000	1.955000
C	-0.696000	6.337000	-7.090000	C	0.325000	-3.890000	0.843000
H	3.672000	5.173000	-6.996000	C	0.459000	-4.731000	-0.448000
H	-1.814000	8.184000	-6.958000	C	2.878000	2.252000	1.597000
H	4.506000	0.817000	-6.926000	C	3.231000	1.567000	2.950000
H	7.488000	0.129000	-6.788000	C	2.338000	0.324000	3.168000
H	-0.633000	5.415000	-6.493000	C	2.472000	-0.618000	1.937000
C	6.595000	0.638000	-6.397000	C	2.253000	0.141000	0.601000
C	-1.682000	7.262000	-6.372000	C	2.703000	-0.693000	-0.621000
H	5.482000	6.431000	-6.363000	O	-1.535000	5.725000	3.610000
H	3.077000	7.246000	-6.351000	O	0.825000	4.093000	4.036000
C	6.889000	2.153000	-6.309000	O	1.528000	2.660000	1.517000
O	-2.886000	6.531000	-6.292000	O	0.050000	5.802000	0.332000
O	9.303000	1.960000	-6.084000	O	1.143000	4.603000	-2.082000
H	6.724000	-2.911000	-5.893000	O	-6.750000	4.965000	1.663000
H	-3.546000	7.054000	-5.853000	O	-4.165000	5.665000	2.766000
H	6.015000	2.632000	-5.844000	O	-1.965000	5.066000	0.905000
O	2.414000	9.091000	-5.687000	O	-4.805000	5.574000	-1.362000
C	1.034000	7.176000	-5.683000	O	-2.416000	7.225000	-1.943000
H	3.294000	9.440000	-5.612000	O	-9.521000	0.588000	-0.374000
H	10.069000	2.231000	-5.592000	O	-8.457000	3.134000	0.484000
H	8.228000	3.677000	-5.578000	O	-5.804000	3.714000	-0.670000
C	2.452000	7.680000	-5.557000	O	-7.233000	1.379000	-3.107000
C	8.124000	2.583000	-5.524000	O	-6.191000	3.950000	-4.191000
O	6.018000	-2.779000	-5.272000	O	-8.034000	-4.734000	0.112000
H	7.987000	-0.986000	-5.165000	O	-9.169000	-2.142000	-0.436000
H	0.965000	6.217000	-5.149000	O	-7.187000	-0.333000	-1.697000
O	6.202000	0.048000	-5.138000	O	-6.074000	-3.665000	-2.769000
O	0.119000	8.104000	-5.096000	O	-4.402000	-1.963000	-4.400000
C	-1.207000	7.612000	-4.968000	O	-3.654000	-5.248000	3.573000
H	-3.187000	5.263000	-4.760000	O	-6.086000	-4.878000	2.036000

H	2.863000	7.404000	-4.575000	O	-5.364000	-4.182000	-0.786000
H	-1.878000	8.355000	-4.512000	O	-2.608000	-6.353000	0.269000
C	7.142000	-0.791000	-4.488000	O	-4.165000	-7.646000	-1.815000
H	8.013000	2.277000	-4.473000	O	0.997000	-2.362000	4.684000
O	-1.168000	6.394000	-4.202000	O	-1.178000	-4.253000	4.301000
C	6.521000	-2.123000	-4.113000	O	-1.867000	-4.627000	1.458000
O	-3.614000	5.126000	-3.922000	O	1.658000	-3.666000	1.303000
H	7.291000	-2.747000	-3.635000	O	0.845000	-6.060000	-0.259000
H	4.590000	-1.315000	-3.593000	O	3.072000	2.535000	3.968000
O	7.615000	-0.104000	-3.329000	O	2.807000	-0.303000	4.342000
H	-1.963000	4.207000	-3.111000	O	1.418000	-1.580000	2.012000
C	5.380000	-1.910000	-3.111000	O	2.973000	1.376000	0.512000
C	-1.862000	6.343000	-2.970000	O	3.993000	-1.257000	-0.515000
C	-2.650000	5.044000	-2.916000	H	-1.232000	7.030000	1.209000
H	-2.547000	7.197000	-2.863000	H	0.321000	6.389000	3.032000
H	0.459000	7.867000	-2.701000	H	-0.573000	3.409000	2.687000
O	4.854000	-3.148000	-2.647000	H	2.175000	4.654000	1.838000
H	5.907000	0.883000	-2.583000	H	-0.113000	3.764000	-0.094000
H	-4.635000	3.557000	-2.276000	H	2.324000	5.601000	-0.691000
C	6.633000	0.117000	-2.274000	H	2.489000	3.820000	-0.723000
H	4.151000	-2.983000	-2.030000	H	-6.751000	5.579000	-1.017000
H	-0.109000	5.557000	-2.015000	H	-5.792000	6.660000	1.006000
C	5.895000	-1.157000	-1.895000	H	-4.218000	4.007000	1.538000
C	-0.844000	6.358000	-1.847000	H	-3.144000	6.786000	0.576000
C	-0.116000	7.697000	-1.779000	H	-3.370000	4.097000	-1.024000
H	-0.836000	8.517000	-1.640000	H	-1.540000	5.342000	-1.928000
O	-3.996000	3.567000	-1.573000	H	-2.922000	5.658000	-3.026000
C	-3.312000	4.825000	-1.567000	H	-8.789000	0.161000	-2.991000
H	-2.707000	1.880000	-1.435000	H	-9.493000	2.159000	-1.694000
H	-4.043000	5.632000	-1.412000	H	-7.074000	1.619000	0.213000
H	6.580000	-1.811000	-1.336000	H	-7.518000	3.899000	-1.884000
O	7.615000	2.000000	-1.321000	H	-5.507000	1.496000	-1.947000
O	4.757000	-0.824000	-1.076000	H	-4.511000	3.278000	-3.176000
C	7.409000	0.620000	-1.047000	H	-5.264000	2.225000	-4.417000
H	8.369000	0.092000	-0.947000	H	-6.481000	-5.463000	-2.051000
H	-0.498000	1.436000	-0.791000	H	-8.618000	-4.250000	-1.808000
O	-2.572000	1.236000	-0.750000	H	-7.159000	-2.211000	0.055000
O	0.756000	7.611000	-0.662000	H	-8.186000	-1.770000	-2.872000
H	8.095000	2.397000	-0.604000	H	-5.223000	-1.972000	-1.887000
O	-1.529000	6.151000	-0.593000	H	-6.440000	-2.046000	-4.761000
H	1.238000	8.425000	-0.572000	H	-5.746000	-0.579000	-3.997000
O	-1.346000	3.830000	-0.566000	H	-1.862000	-6.656000	2.074000
H	2.350000	-1.160000	-0.565000	H	-4.343000	-6.759000	2.362000
C	-2.255000	4.931000	-0.458000	H	-4.450000	-3.760000	1.496000
H	6.819000	0.486000	-0.128000	H	-5.488000	-6.205000	-0.146000
C	-1.322000	1.511000	-0.066000	H	-2.898000	-4.629000	-0.843000
O	3.907000	-3.259000	-0.055000	H	-3.993000	-5.803000	-2.709000
H	3.646000	1.301000	0.033000	H	-2.501000	-6.699000	-2.284000
H	4.749000	-3.676000	0.086000	H	2.881000	-2.964000	2.675000
H	-1.851000	-1.040000	0.122000	H	1.372000	-4.227000	4.112000

C	4.953000	-1.121000	0.297000	H	-1.085000	-2.662000	2.963000
H	5.911000	-1.643000	0.435000	H	-0.138000	-5.559000	2.230000
C	2.492000	-1.327000	0.513000	H	-0.147000	-2.908000	0.557000
H	-2.766000	4.902000	0.516000	H	-0.495000	-4.677000	-1.011000
C	-1.295000	2.933000	0.525000	H	1.261000	-4.328000	-1.094000
O	-1.029000	-0.840000	0.554000	H	3.606000	3.057000	1.340000
H	0.820000	3.153000	0.594000	H	4.312000	1.262000	2.914000
C	3.840000	-2.035000	0.712000	H	1.269000	0.634000	3.310000
O	1.326000	0.682000	0.780000	H	3.478000	-1.113000	1.949000
H	0.603000	-1.724000	0.887000	H	1.164000	0.371000	0.473000
O	4.979000	0.132000	1.002000	H	2.078000	-1.604000	-0.691000
O	1.437000	-2.161000	1.010000	H	2.585000	-0.112000	-1.556000
C	3.751000	0.867000	1.038000	H	-1.276000	5.346000	4.444000
C	-1.061000	0.503000	1.043000	H	1.488000	3.402000	3.988000
H	-2.163000	3.067000	1.187000	H	1.820000	4.786000	-2.721000
C	2.487000	0.003000	1.206000	H	-6.557000	5.074000	2.585000
H	3.618000	6.375000	1.208000	H	-3.251000	5.562000	3.037000
H	-0.189000	5.349000	1.289000	H	-1.813000	7.423000	-1.237000
O	4.595000	3.073000	1.292000	H	-9.794000	1.080000	0.391000
C	-0.006000	3.196000	1.319000	H	-7.821000	3.691000	0.933000
C	0.257000	0.831000	1.738000	H	-6.169000	4.694000	-3.601000
H	3.953000	-2.266000	1.781000	H	-8.876000	-4.462000	0.456000
H	-1.868000	0.598000	1.785000	H	-9.216000	-1.191000	-0.335000
H	4.667000	3.836000	1.854000	H	-4.319000	-2.905000	-4.480000
O	3.945000	5.826000	1.911000	H	-4.496000	-5.009000	3.939000
C	3.849000	2.066000	1.954000	H	-6.734000	-4.619000	1.379000
C	0.082000	4.552000	1.997000	H	-4.956000	-7.497000	-1.303000
H	2.841000	2.441000	2.185000	H	0.196000	-1.831000	4.684000
H	1.418000	6.750000	2.195000	H	-2.069000	-4.472000	4.026000
O	0.207000	2.165000	2.280000	H	0.114000	-6.515000	0.142000
H	2.388000	-0.198000	2.283000	H	3.271000	2.098000	4.787000
O	1.447000	4.708000	2.422000	H	2.247000	-1.069000	4.494000
H	0.401000	0.111000	2.557000	O	9.196000	0.073000	-0.680000
H	-0.593000	4.587000	2.865000	C	7.956000	-0.635000	-0.642000
H	4.356000	1.780000	2.887000	C	7.017000	0.124000	0.332000
C	1.736000	5.990000	2.924000	O	5.729000	-0.483000	0.330000
C	3.246000	6.135000	3.124000	C	5.075000	-0.453000	-0.922000
H	3.449000	7.181000	3.397000	C	5.891000	-1.188000	-2.031000
H	5.628000	5.349000	3.553000	C	7.338000	-0.622000	-2.071000
H	3.643000	4.196000	3.956000	O	8.062000	-1.434000	-2.970000
O	1.033000	6.199000	4.157000	O	5.214000	-0.923000	-3.242000
C	3.778000	5.251000	4.237000	C	7.520000	0.034000	1.790000
O	5.183000	5.556000	4.366000	O	6.884000	0.953000	2.633000
H	1.316000	4.271000	4.870000	C	10.355000	-0.731000	-0.661000
C	1.439000	5.311000	5.205000	O	10.610000	-0.930000	-2.035000
C	2.948000	5.543000	5.490000	C	11.491000	0.024000	-2.629000
H	3.094000	6.595000	5.777000	C	12.912000	-0.088000	-2.007000
H	-0.469000	5.285000	6.229000	O	13.644000	1.104000	-2.204000
C	0.581000	5.507000	6.469000	C	12.866000	-0.309000	-0.464000
O	3.343000	4.677000	6.565000	C	11.469000	0.037000	0.112000

O	0.678000	6.864000	6.954000	O	11.374000	-0.188000	1.493000
H	0.932000	4.819000	7.252000	O	13.134000	-1.662000	-0.149000
H	2.821000	4.869000	7.335000	C	11.497000	-0.310000	-4.142000
H	0.142000	6.962000	7.732000	O	12.056000	0.727000	-4.903000
O	-1.729000	1.949000	-2.770000	H	8.105000	-1.688000	-0.293000
O	0.651000	5.509000	-3.302000	H	6.924000	1.194000	0.019000
O	0.384000	1.457000	-2.544000	H	4.810000	0.590000	-1.227000
O	0.539000	-0.594000	-7.640000	H	5.912000	-2.290000	-1.842000
O	-1.319000	0.528000	-7.816000	H	7.328000	0.424000	-2.470000
N	7.108000	5.679000	-2.767000	H	8.991000	-1.231000	-2.848000
N	1.156000	3.558000	-4.395000	H	5.669000	-1.413000	-3.914000
N	9.758000	5.113000	-4.922000	H	7.421000	-0.997000	2.185000
N	7.958000	4.377000	-6.958000	H	8.583000	0.329000	1.857000
N	9.614000	4.813000	-8.605000	H	5.955000	0.738000	2.657000
N	11.828000	5.600000	-7.941000	H	10.176000	-1.765000	-0.276000
N	12.506000	5.801000	-5.723000	H	11.080000	1.053000	-2.465000
N	11.314000	5.134000	-10.215000	H	13.461000	-0.936000	-2.491000
C	-0.236000	3.045000	-4.344000	H	13.718000	1.230000	-3.141000
C	-0.607000	2.351000	-5.664000	H	13.627000	0.338000	0.043000
C	5.721000	5.447000	-3.075000	H	11.311000	1.141000	0.051000
C	0.231000	1.123000	-5.983000	H	11.592000	-1.097000	1.662000
C	8.094000	4.651000	-3.193000	H	14.063000	-1.788000	-0.281000
C	2.981000	4.967000	-3.467000	H	12.149000	-1.172000	-4.375000
C	1.525000	4.734000	-3.662000	H	10.477000	-0.553000	-4.501000
C	5.233000	4.134000	-3.199000	H	11.456000	1.461000	-4.872000
C	4.809000	6.512000	-3.141000	O	-3.073000	1.482000	8.342000
C	-0.448000	2.095000	-3.167000	O	-0.740000	0.163000	4.960000
C	3.882000	3.902000	-3.394000	O	-4.302000	1.532000	6.541000
C	3.456000	6.273000	-3.332000	O	-3.630000	6.971000	6.131000
C	8.516000	4.727000	-4.636000	O	-1.999000	6.562000	7.515000
C	7.610000	7.065000	-2.888000	N	-3.062000	-0.386000	-1.066000
C	-0.284000	0.385000	-7.192000	N	-1.987000	2.084000	4.877000
C	7.609000	4.349000	-5.684000	N	-3.417000	1.764000	-3.758000
C	10.159000	5.142000	-6.244000	N	-2.443000	3.895000	-2.196000
C	9.258000	4.779000	-7.278000	N	-1.881000	5.103000	-4.163000
C	11.488000	5.543000	-6.634000	N	-2.309000	4.135000	-6.363000
C	10.886000	5.207000	-8.889000	N	-3.420000	2.113000	-6.681000
H	-0.923000	3.923000	-4.218000	N	-1.445000	6.337000	-6.131000
H	-0.522000	3.084000	-6.493000	C	-1.953000	2.297000	6.345000
H	-1.680000	2.074000	-5.622000	C	-1.770000	3.786000	6.673000
H	0.241000	0.418000	-5.125000	C	-2.632000	-0.003000	0.253000
H	1.292000	1.399000	-6.159000	C	-2.907000	4.677000	6.195000
H	1.847000	2.833000	-4.340000	C	-4.036000	0.497000	-1.761000
H	8.965000	4.754000	-2.513000	C	-1.900000	0.576000	2.903000
H	7.658000	3.644000	-3.001000	C	-1.500000	0.862000	4.307000
H	5.925000	3.280000	-3.128000	C	-3.475000	0.761000	1.079000
H	5.163000	7.549000	-3.026000	C	-1.422000	-0.479000	0.783000
H	3.517000	2.869000	-3.491000	C	-3.206000	1.752000	7.027000
H	2.755000	7.116000	-3.373000	C	-3.111000	1.045000	2.384000
H	8.517000	7.163000	-2.277000	C	-1.064000	-0.194000	2.092000

H	6.858000	7.777000	-2.510000	C	-3.441000	1.709000	-2.427000
H	7.855000	7.348000	-3.923000	C	-2.049000	-0.974000	-1.969000
H	6.583000	4.024000	-5.453000	C	-2.763000	6.093000	6.691000
H	-1.778000	1.353000	-2.030000	C	-2.945000	2.806000	-1.642000
H	0.153000	-1.020000	-8.399000	C	-2.909000	2.894000	-4.369000
H	13.293000	6.302000	-6.065000	C	-2.410000	3.971000	-3.592000
H	12.210000	6.069000	-4.815000	C	-2.854000	3.031000	-5.803000
H	12.099000	5.696000	-10.447000	C	-1.862000	5.152000	-5.524000
H	10.595000	5.135000	-10.901000	H	-1.062000	1.749000	6.752000
				H	-0.814000	4.138000	6.235000
				H	-1.649000	3.889000	7.772000
				H	-3.887000	4.287000	6.542000
				H	-2.962000	4.691000	5.087000
				H	-2.840000	2.399000	4.451000
				H	-4.569000	-0.144000	-2.494000
				H	-4.797000	0.834000	-1.021000
				H	-4.440000	1.126000	0.694000
				H	-0.753000	-1.096000	0.163000
				H	-3.781000	1.646000	3.017000
				H	-0.119000	-0.580000	2.495000
				H	-2.566000	-1.552000	-2.746000
				H	-1.390000	-1.657000	-1.409000
				H	-1.416000	-0.220000	-2.460000
				H	-2.967000	2.767000	-0.543000
				H	-3.890000	1.139000	8.692000
				H	-3.489000	7.841000	6.491000
				H	-3.121000	2.159000	-7.627000
				H	-3.528000	1.192000	-6.326000
				H	-1.147000	6.257000	-7.075000
				H	-0.875000	6.930000	-5.573000

4. DSC/TG

a)

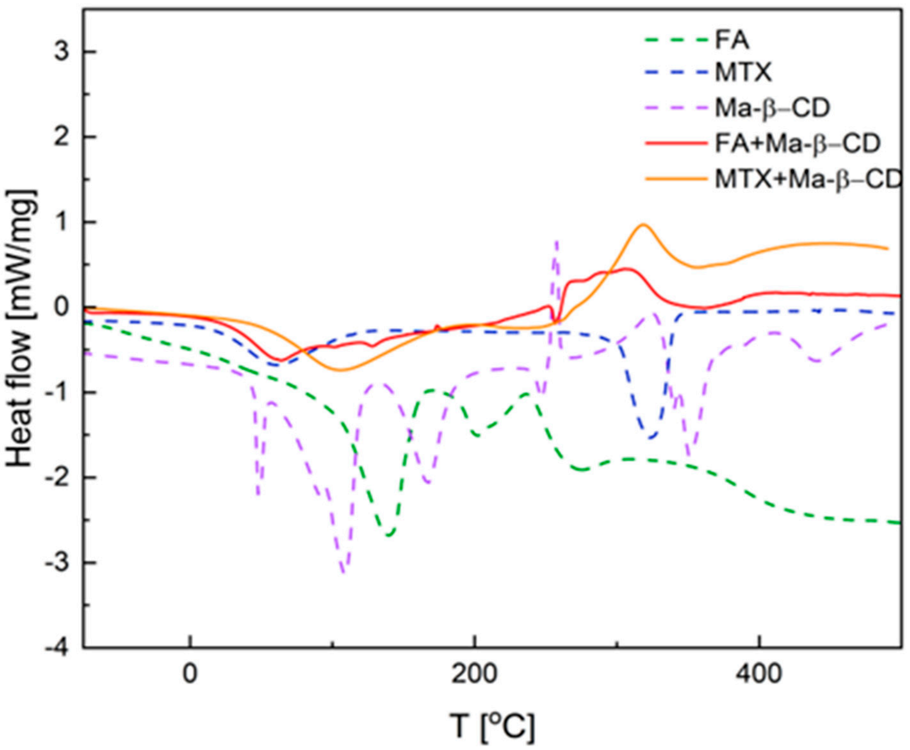

b)

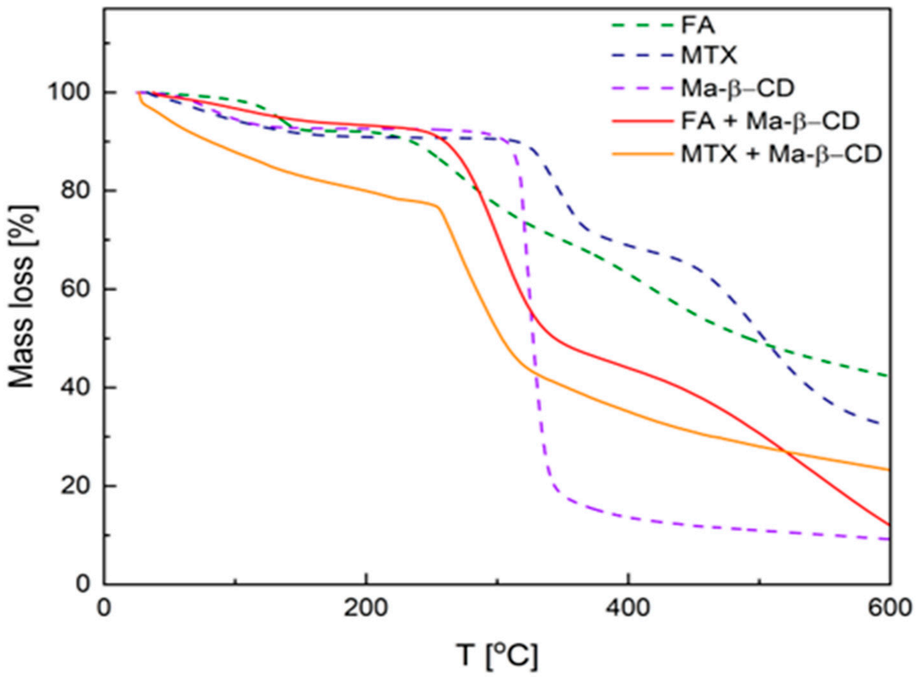

Figure S19. DSC (a) and TGA (b) curves of FA, MTX and their solid state complexes with Ma-β–CD.

5. FTIR

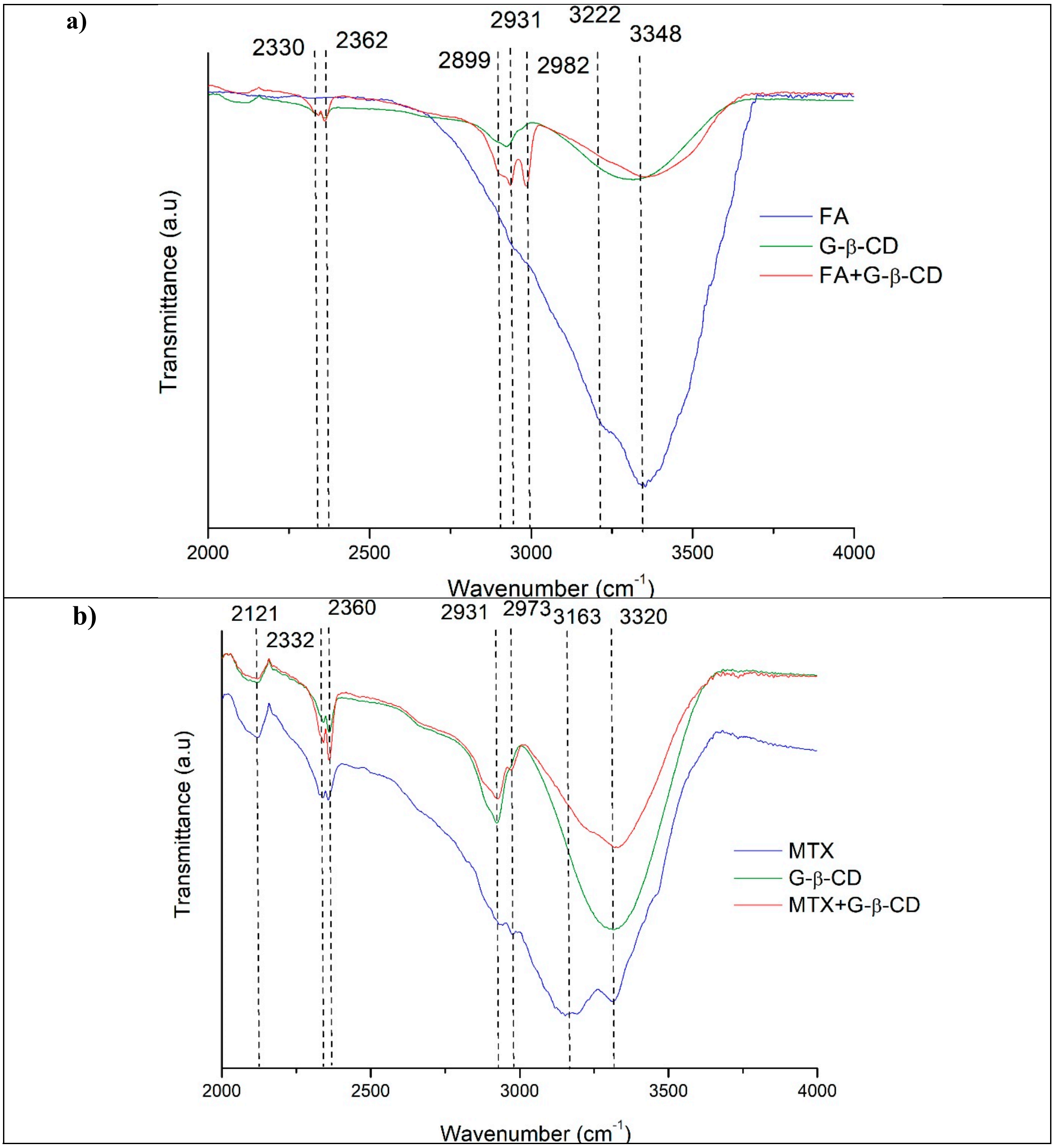

Figure S20. The FTIR spectra in the region 2000-4000 cm^{-1} recorded for: a) FA, G-β–CD, FA/G-β–CD b) MTX, G-β–CD, MTX/G-β–CD.

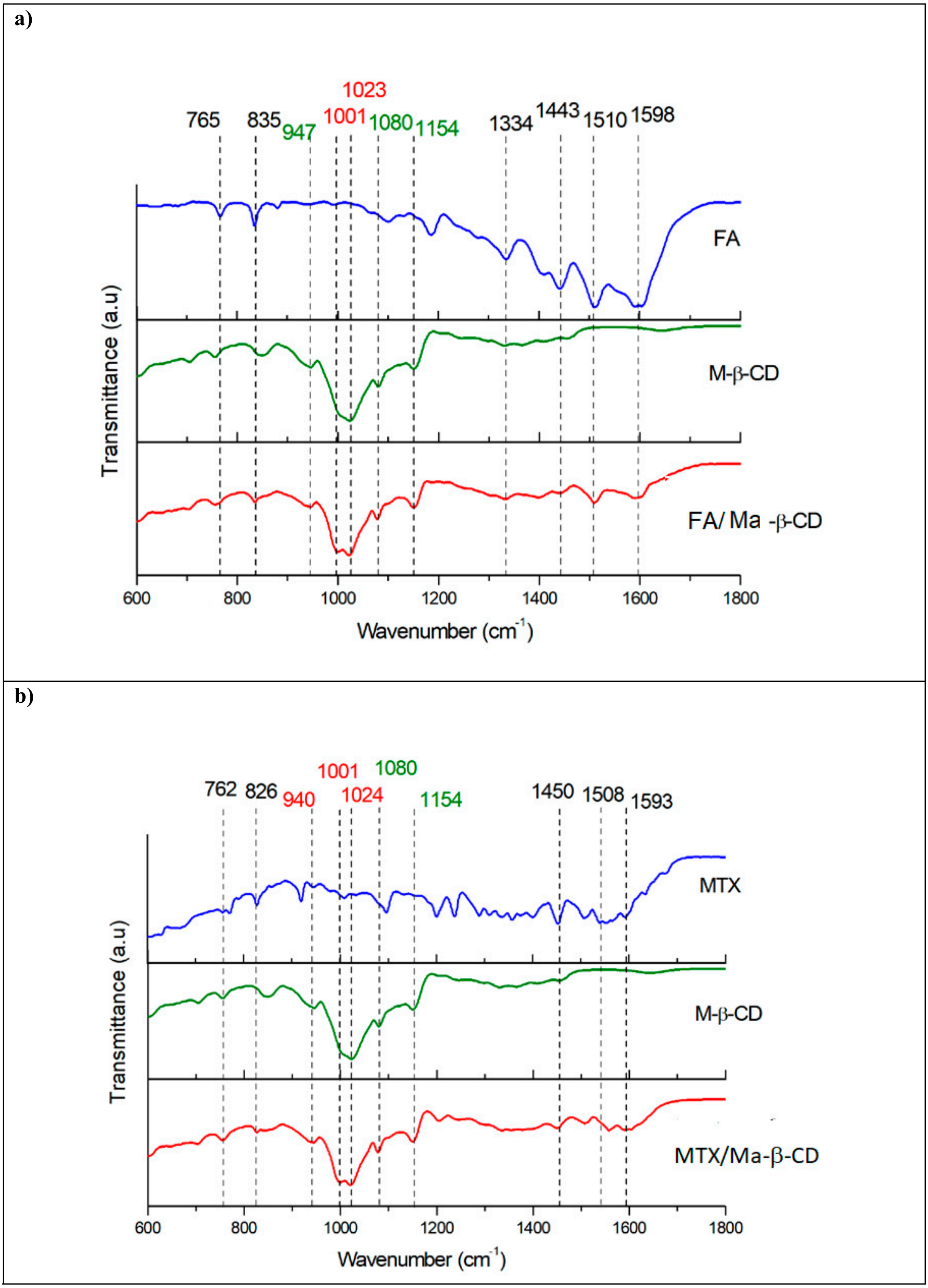

Figure S21. Stacked FTIR spectra: a) FA, Ma-β–CD, FA/Ma-β–CD b) MTX, Ma-β–CD, MTX/Ma-β–CD.

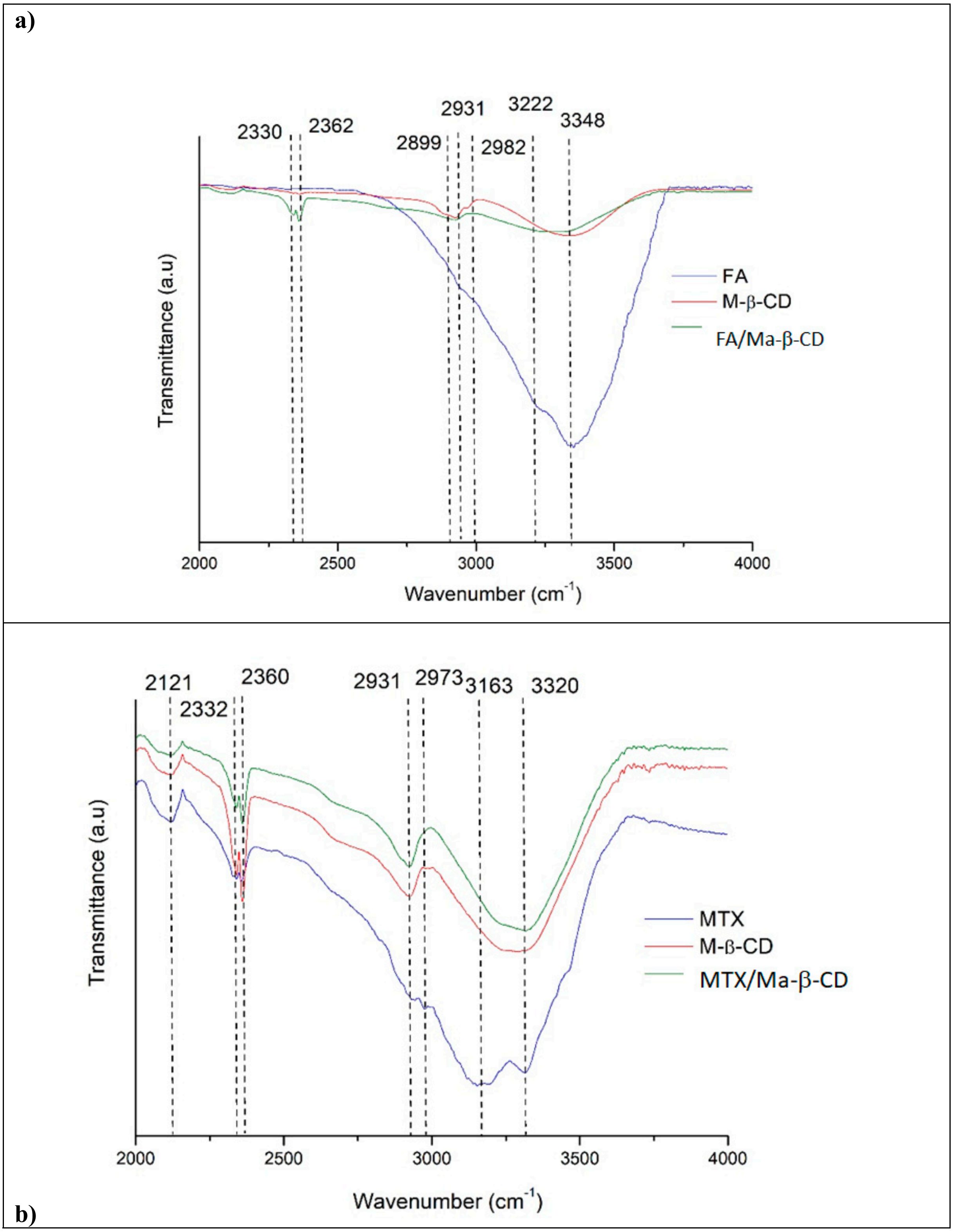

Figure S22. The FTIR spectra in the region 2000-4000 cm^{-1} recorded for: a) FA, Ma-β–CD, FA/Ma-β–CD b) MTX, Ma-β–CD, MTX/Ma-β–CD.

Table S7. The FTIR wavenumbers assignment in sequence: FA, MTX, G-β–CD, FA/G-β–CD, MTX/G-β–CD, Ma-β–CD, FA/Ma-β–CD, MTX/Ma-β–CD. Only bands with discussed changes due to complex formation are shown.

Wavenumbers observed for Molecules								Assignments
**FA**	**MTX**	**G-β-CD**	**FA/ G-β-CD**	**MTX/ G- β–CD**	**Ma-β-CD**	**FA/ Ma-β-CD**	**MTX/ Ma- β–CD**	
765	762	755	765	760	757	757	726	$(COO)^-$
835	826	850	835	826	835	835	826	Skeletal "C–C" stretching, CH out of plane bending (aromatic and skeletal)
879			879					CH_3 and CH out of plane bending of aromatic CCH and skeletal CCH
918	918	933	918	918	947	947	940	"C=O" stretching, in plane bending of CCH
1001	1001	997	1001	1001	1001	1001	1001	Ring deformation
	1025	1021	1034	1025	1023	1023	1154	"C=O" stretching, in plane bending of CCH
		1080	1080		1080	1080		"C–O", "C–C" stretching, in plane bending of OCH
		1154	1154		1154	1154		In plane bending CCH (aromatic and skeletal), "C–O" symmetric stretching
	1199			1208				rocking (NH_3^+), rocking (CH)(OH), ring deformations
	1240			1250				Stretching (C–N), bending (N–H),
	1290							CH in plane bending of "C=CH", aromatic "C–O" stretching
1334						1334		"C–O–C" stretching, out of

						plane bending of CH_3, in plane bending of aromatic CCH
1443	1450	1443	1450	1443	1450	In plane bending of CH
1510	1508	1510	1508	1510		“C=C” stretching, in plane bending of CH_3, “C=O” stretching
1598	1593	1598	1593	1598		“C–C=O” in plane bending
